# Deep brain stimulation for Parkinson’s disease: bibliometric analysis of the top 100 cited literature

**DOI:** 10.3389/fnagi.2024.1413074

**Published:** 2024-10-16

**Authors:** Weijie Zhao, Xinxin Shao, Ziyue Wang, Chuanhao Mi, Yu Wang, Xianghua Qi, Xiao Ding

**Affiliations:** ^1^Department of First Clinical Medical College, Shandong University of Traditional Chinese Medicine, Jinan, China; ^2^College of Traditional Chinese Medicine, Shandong University of Traditional Chinese Medicine, Jinan, China; ^3^Department of Ophthalmology, Beijing Friendship Hospital, Capital Medical University, Beijing, China; ^4^Affiliated Hospital of Shandong University of Traditional Chinese Medicine, Jinan, China

**Keywords:** deep brain stimulation, Parkinson’s disease, bibliometrics, research trends, visualized study, top-cited

## Abstract

**Background:**

Deep Brain Stimulation (DBS) has been widely applied and accepted in the treatment of neurological and psychiatric disorders. Despite numerous studies exploring the effects of DBS on the progression of neurodegenerative diseases and the treatment of advanced Parkinson’s disease (PD), there is a limited number of articles summarizing this research. The purpose of this study is to investigate the current trends, hot topics, and potential in research surrounding DBS therapy for PD, as well as to anticipate the challenges of such research.

**Methods:**

We searched the Web of Science Core Collection database (WoSCC) for DBS research literature related to PD published from January 2014 to January 2024, utilized CiteSpace, VOS viewer, the bibliometric online analysis platform, Scimago Graphica, Microsoft Excel 2021, and R software version 4.2.3 for data analysis. And we conducted quantitative research on publications, citations, journals, authors, countries, institutions, keywords, and references, visualized the results in network graphs.

**Results:**

From 2014 to 2024, papers from 39 journals from 11 countries were among the top 100 cited. Most papers were published in Neurology, with the highest average citations per paper in Nature Neuroscience. The United States (US) contributed the most publications, followed by the United Kingdom (UK) and Germany. In terms of total publications, University College London (UCL) contributed the most papers. The primary classifications of articles were Clinical Neurology, Neurosciences, and Surgery. The top five keywords were subthalamic nucleus, DBS, PD, medical therapy, and basal ganglia. Cluster analysis indicates that DBS research focus on improving quality of life and applying computational models.

**Conclusion:**

Through bibliometric analysis, researchers could quickly and clearly understand the hotspots and boundaries of their research field, thus guiding their research direction and scope to improve research efficiency and the quality of outcomes. Although studies indicate that DBS is currently a crucial method for treating advanced PD, in the long run, creating a personalized, low-cost treatment regimen with precise targeting and long-term efficacy poses a challenge.

## 1 Introduction

Parkinson’s disease (PD) is a chronic progressive neurodegenerative disorder that typically manifests in middle to late adulthood but can also affect younger individuals. Globally, at least 5.8 million people are afflicted by this disease (Vos et al., 2020). This disease is primarily caused by the degeneration and death of dopaminergic neurons in the substantia nigra region of the brain. The exact reasons and mechanisms underlying cell death remain unclear, but research suggests that they may be linked to various factors, including genetics (Belfiori et al., 2024), the environment (De Miranda et al., 2022; Gunnarsson and Bodin, 2019), oxidative stress, and neuroinflammation (Song et al., 2024). Clinical symptoms mainly manifest in two major categories: motor symptoms and nonmotor symptoms. Motor symptoms include bradykinesia, increases in muscle rigidity, resting tremor, and postural instability, while nonmotor symptoms include sleep disturbances; psychiatric symptoms such as depression and anxiety; autonomic nervous system symptoms such as constipation and hypotension; and sensory disturbances such as numbness and restless legs syndrome. These symptoms significantly impact patients’ daily lives, leading to decreased self-care abilities, impaired work and social functioning, and due to the incurable nature of the disease and the rising rate of diagnosis, highlighting the increasing healthcare demands (Dorsey et al., 2018; Schiess et al., 2022). Researchers are diligently working to explore various possible methods and strategies to find more effective treatment options for PD (Höglinger et al., 2024). Unfortunately, it cannot currently be cured, but symptoms can be alleviated, functional disabilities can be reduced, and disease progression can be delayed through methods such as medication, surgical treatment, stem cell therapy, and rehabilitation psychology. Drug therapy mainly includes anticholinergic drugs, dopamine agonists, levodopa preparations, and dopamine receptor agonists. Surgical treatments such as deep brain stimulation (DBS) (Deuschl et al., 2006), stem cell therapy (Parmar et al., 2020), and gene therapy (Patel et al., 2024) can help maintain muscle function and daily activities. Medication induced dyskinesia becomes intolerable at severe stages of the disease and DBS most commonly provides motor-symptom relief alongside medication to reduce the prevalence and severity of medication side-effects. Therefore, many patients receive a reduction in their dopaminergic medication when paired with DBS. In recent years, an increasing number of PD patients have undergone DBS treatment (Rowland et al., 2016).

DBS is a surgical intervention method first utilized for PD treatment by Benabid Al et al. (1987). It involves implanting electrodes in specific brain regions and providing electrical stimulation through an external pulse generator, thereby aiming to improve symptoms of neurological disorders. At present, the exact mechanism of DBS treatment for PD remains an actively researched area. A prevailing theory suggests that DBS may work by suppressing pathological oscillatory activity in neural networks associated with PD. In particular, high-frequency stimulation may suppress abnormal beta-band oscillations, which are thought to contribute to the motor symptoms of PD (Bočková et al., 2024). It has now become a conventional treatment method for advanced PD when medication therapy is insufficiently effective, and it can even be used in the early stages (Hacker et al., 2020). It holds significant importance in reducing medication usage, improving quality of life, providing personalized adjustments, reversibility, and adjustability, and offering patients better treatment options and quality of life. In recent years, researchers have made continuous advancements in treatment targets, DBS devices, implantation surgeries, and postoperative programming, enhancing treatment efficacy and reducing the occurrence of DBS motor complications (Bove et al., 2021). However, understanding the overall progress and research trends in this field poses a challenge.

Bibliometric analysis serves as a scientific and efficient exploration method, offering comprehensive support and guidance to researchers in their respective fields of study (Huang et al., 2024; Karabay et al., 2024). It enables researchers to gain in-depth insights into the prevalent issues, key trends, and research boundaries within their field, thereby steering their research direction and fostering the enhancement and assurance of research outcome quality. There is a lack of bibliometric analysis in the field of DBS treatment for PD. Therefore, after obtaining the top 100 cited articles on DBS, we utilized bibliometric analysis to assess the current status, hot topics, and trends in DBS research and to propose plans and recommendations.

## 2 Materials and methods

### 2.1 Data sources and search strategies

The articles for the study were sourced from the SCI-Expanded database within the Web of Science Core Collection (WoSCC). This database includes over 10,000 journals and is widely recognized as one of the most authoritative resources for bibliometric analysis (Chen, 2020). On February 21, 2024, we conducted a search using the following search strategy: TI = (“Parkinson*”) AND TI = (“Deep Brain Stimulation*” OR“Electrical Stimulation of the Brain”). We limited our search to articles published in English within the time frame from January 1, 2014 to January 1, 2024. The document type was restricted to “articles.”

### 2.2 Selection criteria

To determine the top 100 most cited articles, researchers classified publications by citation count, and manually sifted through the raw data to determine the final selection of articles, including reading the paper’s title, abstract, date of publication, and reading the full text when necessary. Inclusion criteria, referencing (Yang et al., 2024), including content and quality criteria, were developed for this study with reference to reviews on related topics and are shown in Table 1.

**Table 1:** Inclusion criteria in manual review process.

Type	Inclusion criteria
Content criteria	Included literature should include research topics in PD.
	Topics related to treating DBS, including surgical treatment, patient selection, and mechanistic studies, should also include treatment outcomes, postoperative management, medical care, and prevention and management of complications.
Quality criteria	All included literature should contain at least two pages of content; literature that does not fulfill this condition will not be considered.
	The document must contain the essential elements of an academic paper, such as an abstract, author information, keywords, and references.
	Literature lacking the above elements will not be considered.
	double-blind peer review

### 2.3 Data selection

We retrieved the parameters and data of the top 100 articles in “Txt” format from WoSCC and then imported them into EndNote for visualization analysis. Throughout this process, we rectified any inaccuracies in the vocabulary and consolidated duplicate terms. This meticulous approach ensured the precision and comprehensiveness of the data, laying a dependable groundwork for subsequent analysis and research.

### 2.4 Bibliometric analysis

To display and analyze the features of the articles, we used CiteSpace 6.3. R1, VOS Viewer 1.6.19, Scimago Graphica 1.0.39, Microsoft Excel 2021, and the bibliometric online analytic platform. This technique sought to determine the present status of research, identify focal points, and reveal new trends in the field of DBS for the treatment of Parkinson’s disease. The top 100 publications were tabulated for analysis using Microsoft Excel 2021. The frequency of inter-country cooperation was calculated in Bibliometrix and can be found at: https://bibliometric.com. The VOS Viewer was used to build a scientometrics network and visualize knowledge (van Eck and Waltman, 2010), including the number of publications per year for journals, authors, countries, and institutions. It can be accessed at can be accessed at: https://www.vosviewer.com/getting-started.CiteSpace allows for a better understanding of emerging hotspots in the field of DBS (Farahat and Elsaid, 2022), assessment of knowledge flows, keyword hotspots, classifications, and research trends, references cited, time slices set to 1 year, g-index (k = 25), LRF = 3.0, L/N = 10, LBY = 5, e = 1.0. It can be accessed and downloaded at: https://citespace.podia.com/. Use Scimago Graphica to visualize the number of publications, citations, and degree of association for journals, authors, countries, and institutions. We performed statistical analyses using R software version 4.2.3^1^ to explore the correlation between the production of articles on DBS for PD and indicators of the size of the economy in each country, which included the Gross Domestic Product (GDP) as well as GDP *per capita* values, obtained from the World Bank website.^2^ As the sample size was less than 50, we checked whether the data were normally distributed using the Shapiro–Wilk test, which was later determined by Spearman correlation analysis. For statistical significance limits, *p* < 0.05 was accepted. And in this study, a “two-stage clustering” strategy was used, in which the data were first preprocessed using spectral clustering to identify underlying structures and clustering trends in the data, and then K-Means clustering was used to improve the representativeness of the cluster centers. After the two-stage clustering was completed, the effectiveness of the clustering was evaluated based on the modularity value (Q) and the average silhouette value (S) provided by the Citespace software.

## 3 Results

### 3.1 Analysis of included citation characteristics

**Figure 1:**
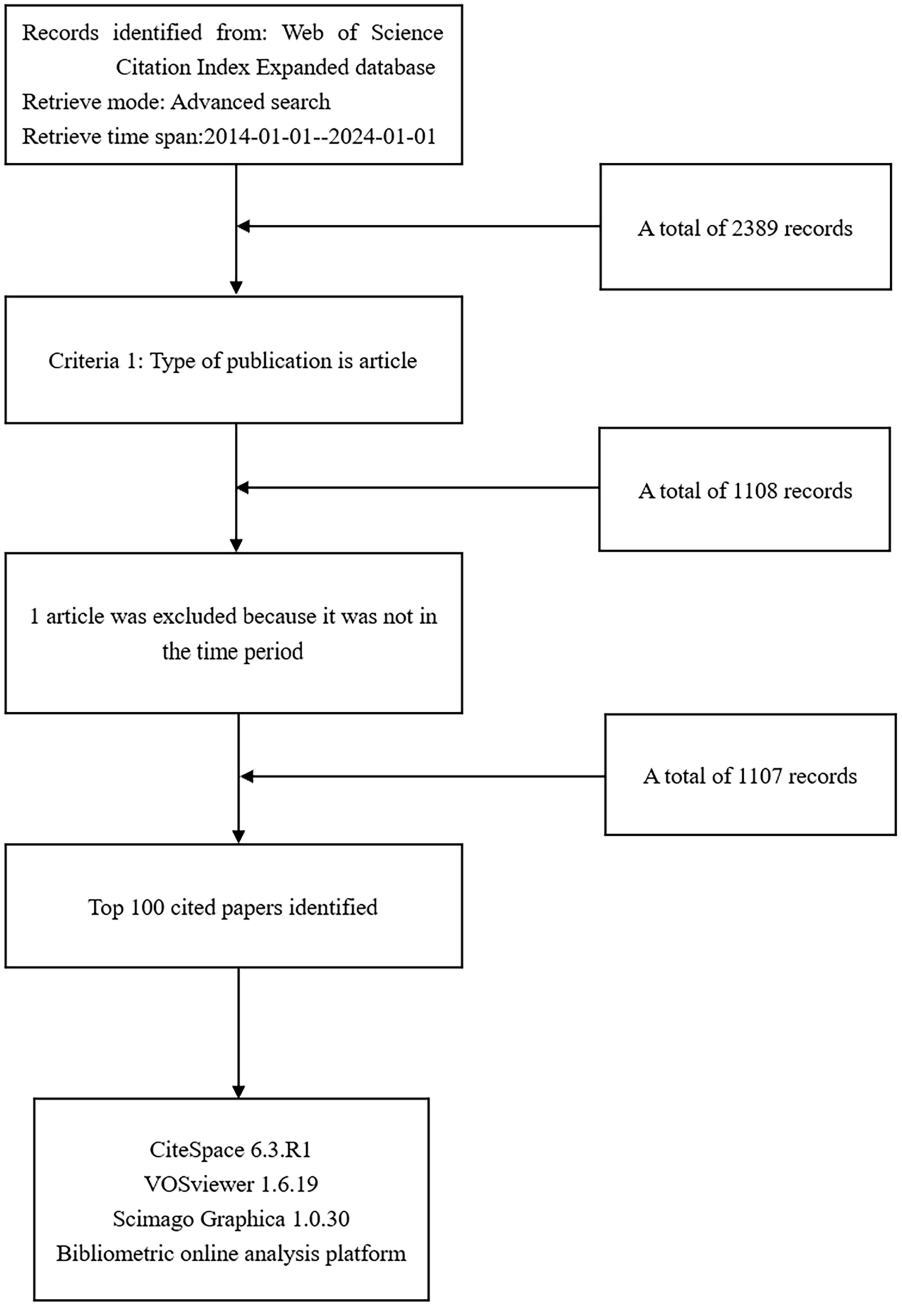
Flow chart of the literature screening process.

As of January 1, 2024, a total of 2,389 studies had been published, of which 1,281 were not articles or reviews. After excluding irrelevant studies, 1,107 studies remained. The outstanding articles were ranked in descending order based on their citation frequency. Figure 1 illustrates the specific process involved.

Supplementary Table S1 lists the top 100 most cited papers, spanning from 2014 to 2021. The total citation count was 8,242 (average = 82.42), with a total of 4,410 references (average = 44.10). The most frequently cited paper was “Therapeutic deep brain stimulation reduces cortical phase-amplitude coupling in Parkinson’s disease” (400 citations) by de Hemptinne and Swann (de Hemptinne et al., 2015), which suggested the possibility of using cortical phase-amplitude coupling as a control signal for closed-loop DBS devices. Following that was “Connectivity predicts Deep Brain Stimulation outcome in Parkinson disease” (394 citations) by Horn and Reich (Horn et al., 2017), who proposed and validated that the therapeutic benefits of DBS on PD might be impacted by the stimulation site and connectivity with other brain regions. The predictive value of this connectivity for treatment outcomes was explored. Additionally, “The modulatory effect of adaptive deep brain stimulation on beta bursts in Parkinson’s disease” (250 citations) by Tinkhauser and Pogosyan (Tinkhauser et al., 2017) proposed and explored the effectiveness of adaptive stimulation in DBS compared to fixed stimulation, which can selectively control excessive beta activity. These aforementioned papers were collectively cited 1,044 times, accounting for one-eighth of the total citations.

### 3.2 Analysis of year of publication and citation

**Figure 2:**
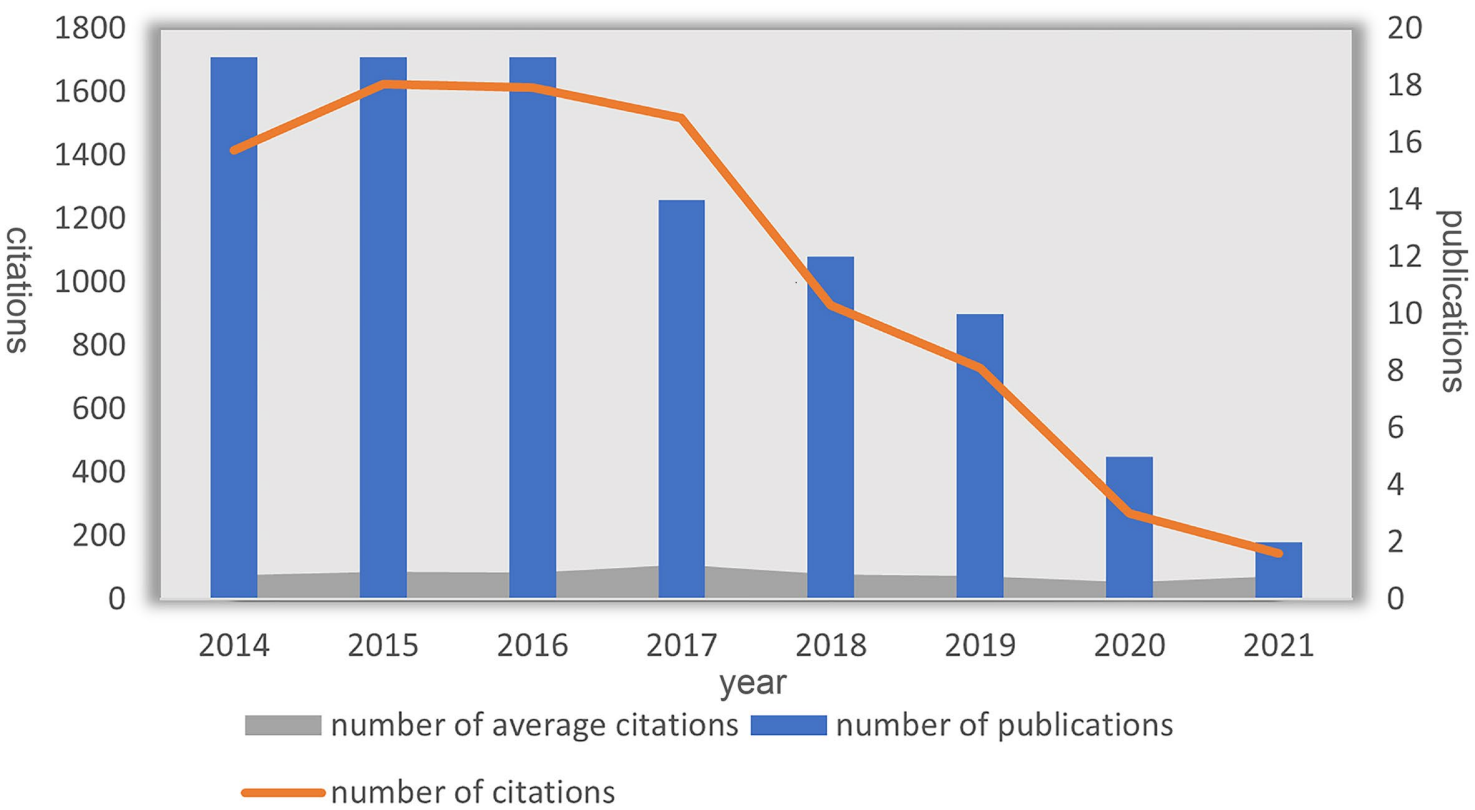
Annual number of publications and citations and average citations for DBS treatment of PD.

Figure 2 depicts the overall distribution of annual citation frequencies and total citations. From 2014 to 2021, the 100 most frequently cited papers were published. Notably, the highest average citation count occurred in 2017, with the same and most numerous publications (*N* = 19) appearing consecutively from 2014 to 2016. DBS research was greatly influenced by the most cited publications during these three years.

### 3.3 Analysis of journal distribution

**Figure 3:**
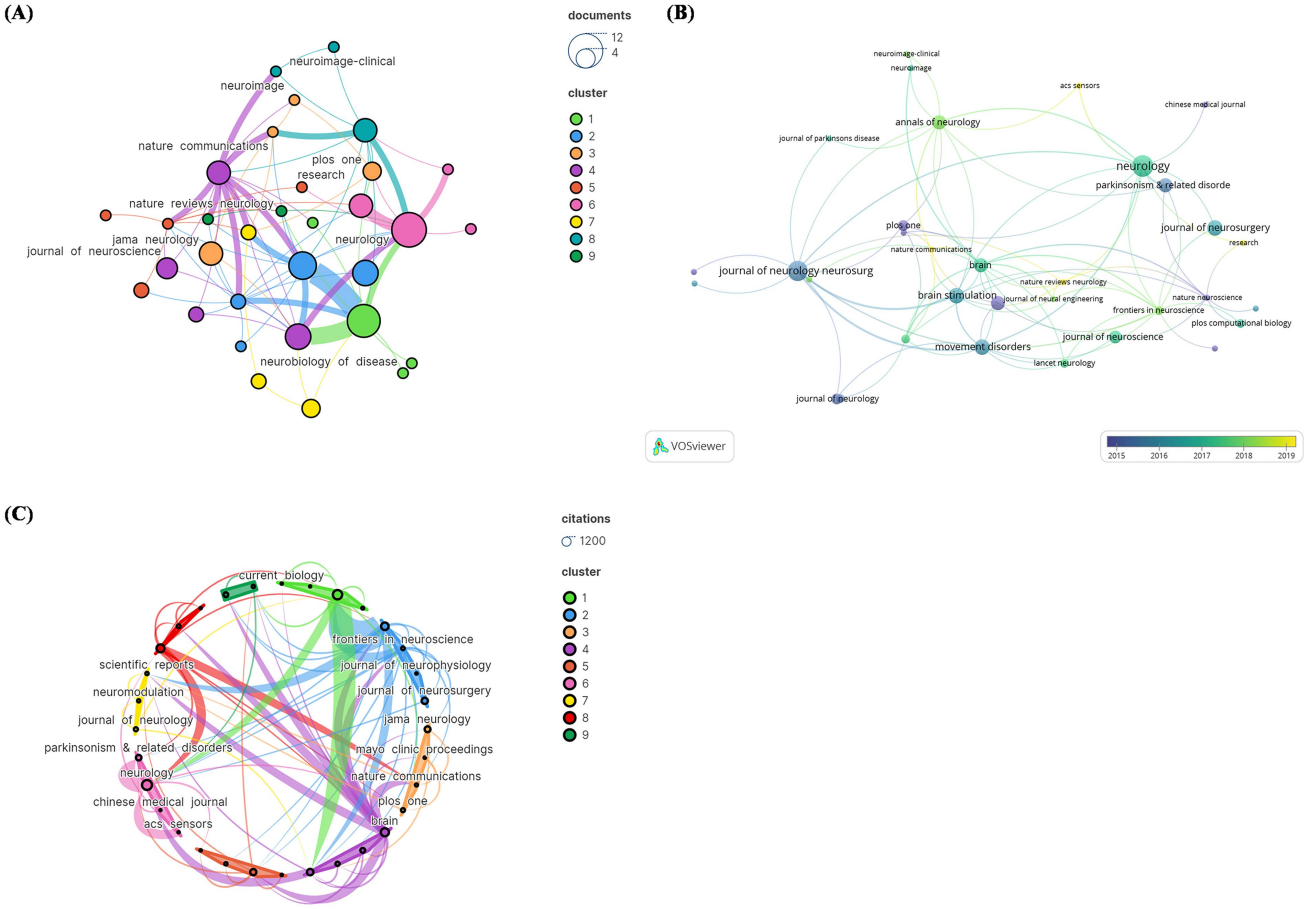
The visual analysis of the journal distribution in the 100 most frequently cited papers. **(A)** Publication count; **(B)** number of publications changing per year; **(C)** overall citation count.

The 100 most commonly referenced pieces appeared in 39 distinct journals. Papers published in Neurology outnumber those in any other journal (*N* = 11), followed by the Journal of Neurology Neurosurgery and Psychiatry (*N* = 10) (Figure 3A). Figure 3B illustrates the number of papers published by journal each year, with ACS Sensors, Nature Communications, and Nature Reviews Neurology being emerging journals in this field. In terms of citation counts, Neurology received the greatest amount of citations (*N* = 1,008). Articles published in high-impact journals such as “Neurology” were more likely to be cited. Conversely, the average citation count was highest for publications in “Nature Neuroscience” (1 article, 400 citations). This graph highlights the influential journals and citations in the field of DBS (Figure 3C).

**Figure 4:**
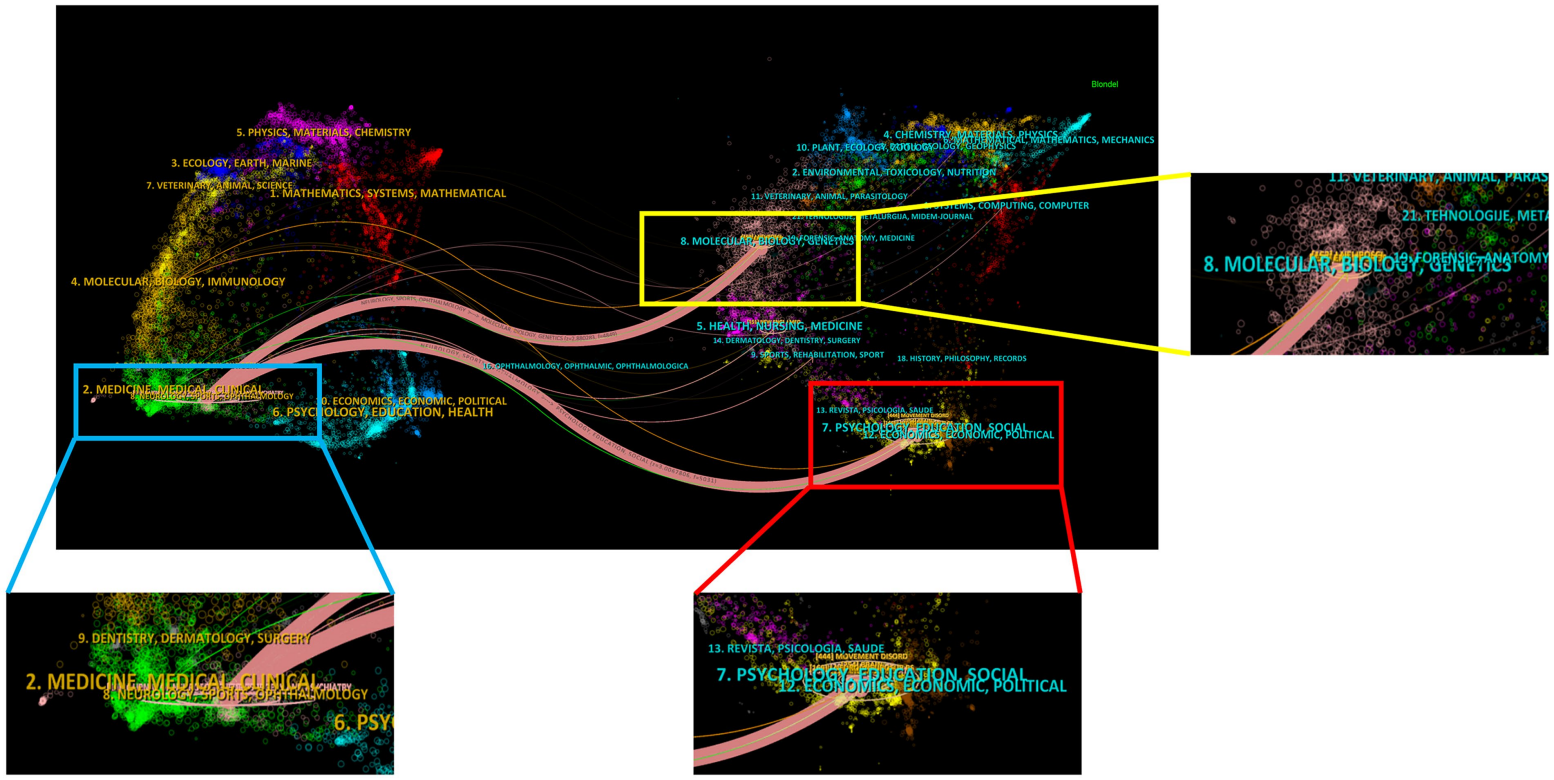
The journals of the 100 most frequently cited studies on DBS treatment for PD patients in a dual map overlay.

Figure 4 shows two primary citation pathways from the dual-map overlay. According to these pathways, papers published in Medicine/Medical/Clinical or Neurology/Sports/Ophthalmology-type journals were built upon by papers in Molecular/Biology/Genetics and Psychology/Education/Social or Economics/Economic/Political-type journals. This conclusion provides guidance for new DBS researchers.

### 3.4 Analysis of authors and coauthors

**Figure 5:**
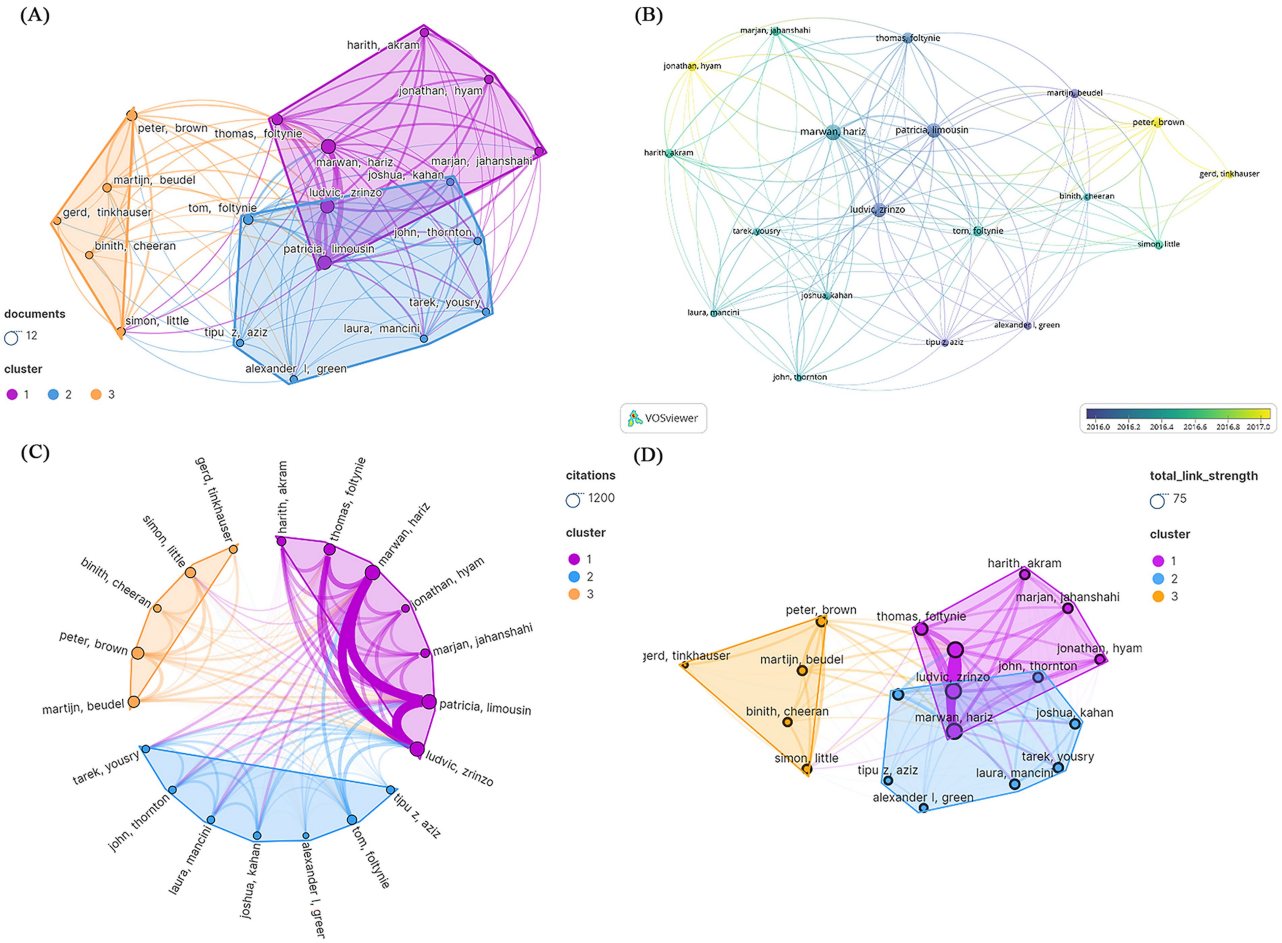
Visualization of the authors of the 100 most frequently cited papers. **(A)** Publication count; **(B)** number of publications changing per year; **(C)** overall citation count; **(D)** total relevance strength.

Among the three different author groups represented by distinct hues, some individuals engaged in active collaboration. The 100 most frequently cited papers were authored by institutions from 524 different entities. Inconsistent modifications were made to author names, such as “Brown, P” being changed to “Peter, Brown “. Hariz, Marwan wrote the most papers (*N* = 11), followed by Limousin, Patricia (*N* = 10) and Zrinzo, Ludvic (*N* = 10). Hariz, Marwan, Limousin, Patricia, Zrinzo, and Ludvic formed the largest group, each with 17 connections (Figure 5A). Figure 5B shows the number of articles published per year. The author with the highest citation count was Hariz, Marwan (*N* = 1,161), followed by Limousin, Patricia (*N* = 1,116) and Zrinzo, Ludvic (*N* = 1,116). However, Brown Peter had the greatest average amount of citations per manuscript (7 papers, 963 citations) (Figure 5C). Limousin, Patricia, is a leading author, while Hyam, Jonathan and Brown, Peter are emerging authors (Figure 5B). Figure 5D depicts the overall connection strength between the authors.

### 3.5 Analysis of countries and institutions

#### 3.5.1 Countries

**Figure 6:**
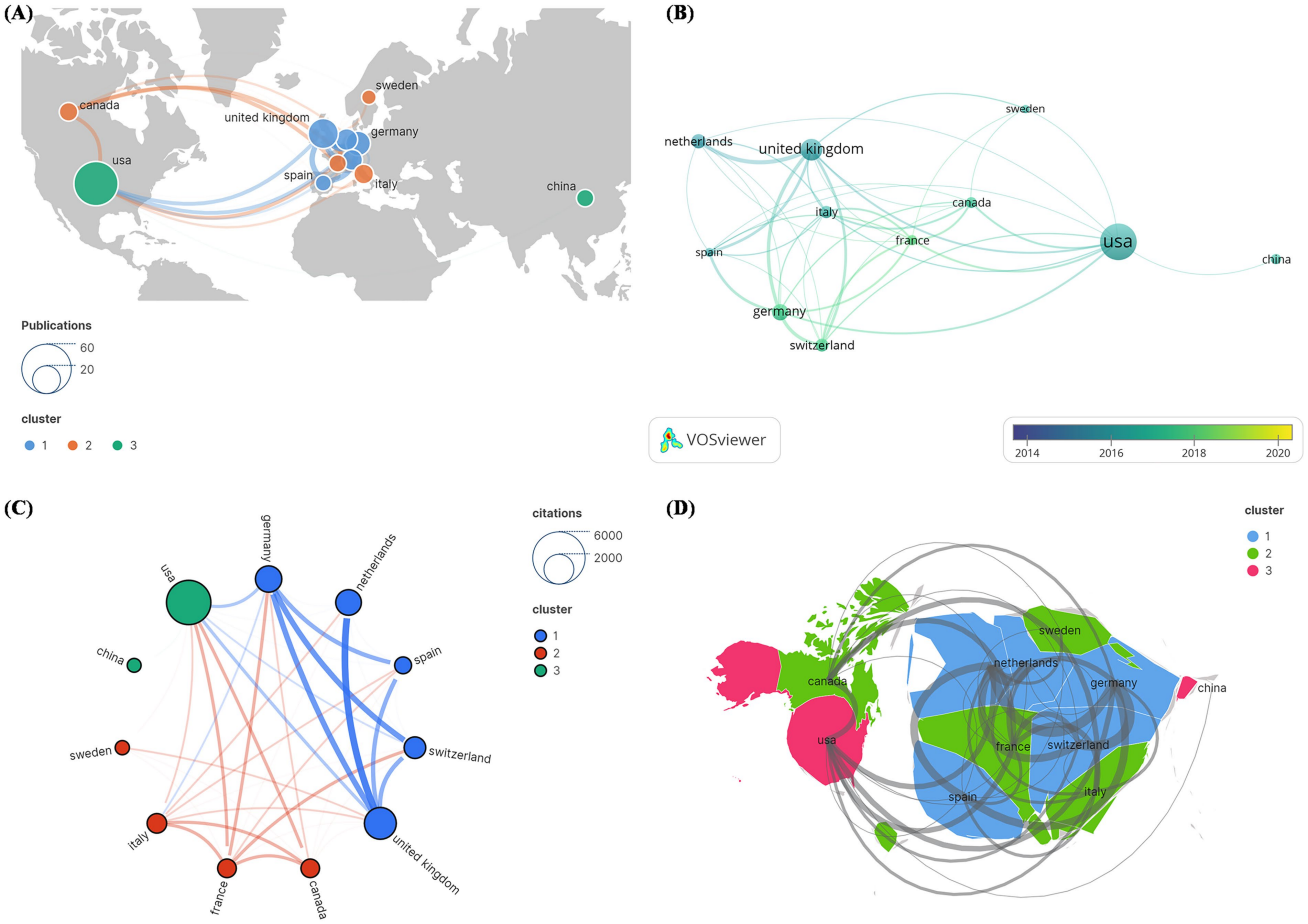
Cocitation network analysis of countries and regions in the 100 most frequently cited papers. **(A)** Publication count; **(B)** number of publications changing annually; **(C)** overall citation count; **(D)** total relevance.

The 100 most frequently cited papers were written by authors from 11 countries. The United States (US) contributed the most publications (*N* = 45), followed by the United Kingdom (UK) (*N* = 21) and Germany (*N* = 14) (Figure 6A). In Figure 6B, the cumulative number of published articles over the years is depicted by country. Among these 11 countries, the US had the highest number of citations (*N* = 4,010), followed by the UK (*N* = 2035) and Germany (*N* = 1,365) (Figure 6C). These statistical data indicate that significant progress has been made by the US in the field of DBS research. The 11 nations were organized into three primary clusters, with the US having the most countries covered by the national cooperation network, intimately connected to all of the other ten, followed by the UK, which had ties to nine countries. The bibliometric map revealed tight links between nations. Thicker lines linking nodes between nations demonstrate international collaboration. Figure 6D depicts the total connection strength between nations. The passing of Resolution WHA73.10 during the 73rd World Health Assembly called for member states to collectively develop a global action plan to address neurological disorders such as PD. The adoption of this resolution inspired efforts toward coordinated cooperation among countries, potentially driving research and exploration into PD in the US, the UK, and France. Notably, the research was predominantly concentrated in developed Western countries, with limited research from developing countries. However, the majority of PD patients reside in low- and middle-income countries, highlighting significant disparities in access to treatment (Schiess et al., 2022).

There was a statistically significant positive correlation between articles published in each country on DBS for PD and GDP (rho = 0.56, *p* = 0.00338). In terms of GDP *per capita*, although there was a slight trend toward a positive correlation (rho = 0.33), this relationship was not statistically significant (*p* = 0.1039).

#### 3.5.2 Institutions

**Figure 7:**
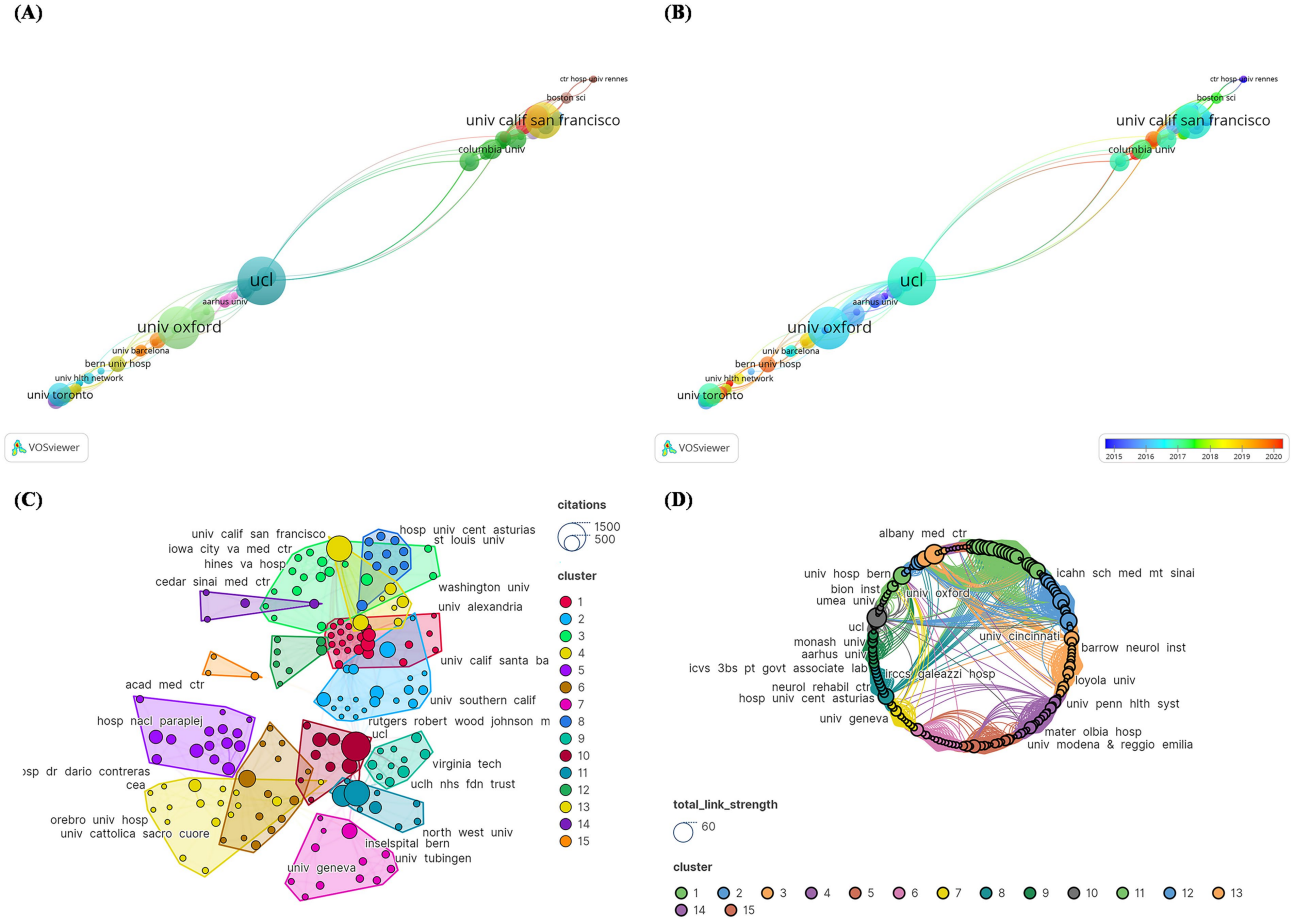
Cocitation network analysis of institutions with the 100 most frequently cited papers. **(A)** publication count; **(B)** number of publications changing annually; **(C)** overall citation count; **(D)** total relevance.

Inconsistencies were addressed in modifying institution names, such as changing “UCL Inst Neurol” to “UCL.” In terms of total publications, University College London (UCL) had the most papers (*N* = 14), followed by Univ Oxford (*N* = 11) and Univ Calif San Francisco (*N* = 9) (Figure 7A). However, in recent years, the University of Cincinnati, Université Grenoble Alpes, and Bern University Hospital have published the most publications (Figure 7B). A total of 237 institutions were divided into 15 primary groups. The institution with the highest number of citations was UCL (*N* = 1,449), followed by Univ Oxford (*N* = 1,122) and Univ Calif San Francisco (*N* = 1,114). The largest institutional collaboration network was composed of Cedars-Sinai Medical Center, covering 22 institutions (Figure 7C). Figure 7D represents the total link strength between institutions, with central institutions predominantly from the US. This indicated that the US serves as a significant collaboration hub in DBS research.

#### 3.5.3 Research focus

According to the WoSCC classification, the 100 most frequently cited papers in DBS belonged to a variety of research topics. The most prominent research topic was “clinical neurology” (*N* = 72), followed by “neurosciences” (*N* = 30), “surgery” (*N* = 22), “psychiatry” (*N* = 10), and “multidisciplinary sciences” (*N* = 7). The 100 most frequently cited papers contained 196 keywords.

**Figure 8:**
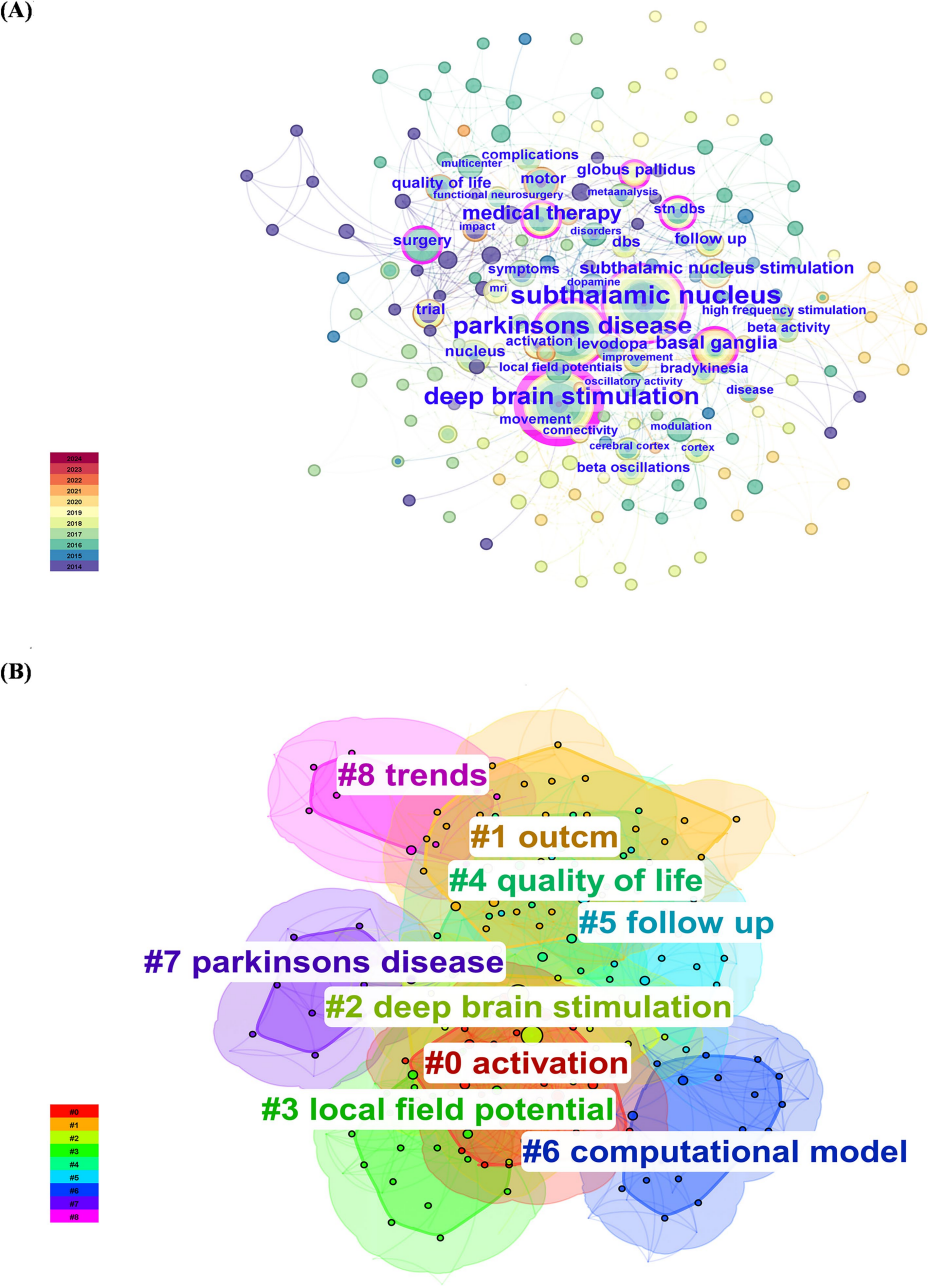
Network visualization and cluster analysis of the 100 most frequently cited papers. **(A)** Keyword co-occurrence; **(B)** keyword clustering.

As shown in Figure 8A and Table 2, the most common keywords were subthalamic nucleus (*N* = 47), deep brain stimulation (*N* = 39), Parkinson’s disease (*N* = 39), medical therapy (*N* = 17), and basal ganglia (*N* = 13). The centrality of deep brain stimulation (0.45) was the highest, followed by that of subthalamic nucleus (0.18) and Parkinson’s disease (0.15). The STN is a popular research target for the treatment of PD. DBS is a therapeutic approach used to alleviate symptoms of PD when there is a decrease in medication efficacy or severe complications. Research on DBS as a therapeutic modality has focused primarily on targets within the basal ganglia (BG), specifically the subthalamic nucleus (STN) and globus pallidus (GP). The mainstream DBS treatment method is subthalamic nucleus stimulation DBS, also known as STN-DBS, which effectively compensates for the limitations in controlling the advanced stages of PD with levodopa. Researchers can identify relevant study information by clustering keywords based on subjects. Generally, a clustering graph was considered acceptable when the modularity value (Q) was greater than 0.3 and the average silhouette value (S) was greater than 0.5. Therefore, based on the results of this experiment (Q = 0.4996, S = 0.7782), it could be concluded that the clustering graph has a high level of credibility. The keywords were divided into the following groups: #0, activation; #1, outcome; #2, deep brain stimulation; #3, local field potential; #4, quality of life; #5, follow-up; #6, computational model; #7, Parkinson’s disease; and #8, trends (Figure 8B).

**Table 2:** Top 10 co-occurring keywords found in the 100 most cited DBS papers.

Rank	Keyword	Occurrences	Centrality
1	Subthalamic nucleus	47	0.18
2	Deep brain stimulation	39	0.45
3	Parkinson’s disease	39	0.15
4	Medical therapy	17	0.12
5	Basal ganglia	13	0.15
6	Subthalamic nucleus stimulation	10	0.05
7	Nucleus	9	0.07
8	Motor	9	0.03
9	Levodopa	8	0.09
10	Globus pallidus	8	0.12

**Figure 9:**
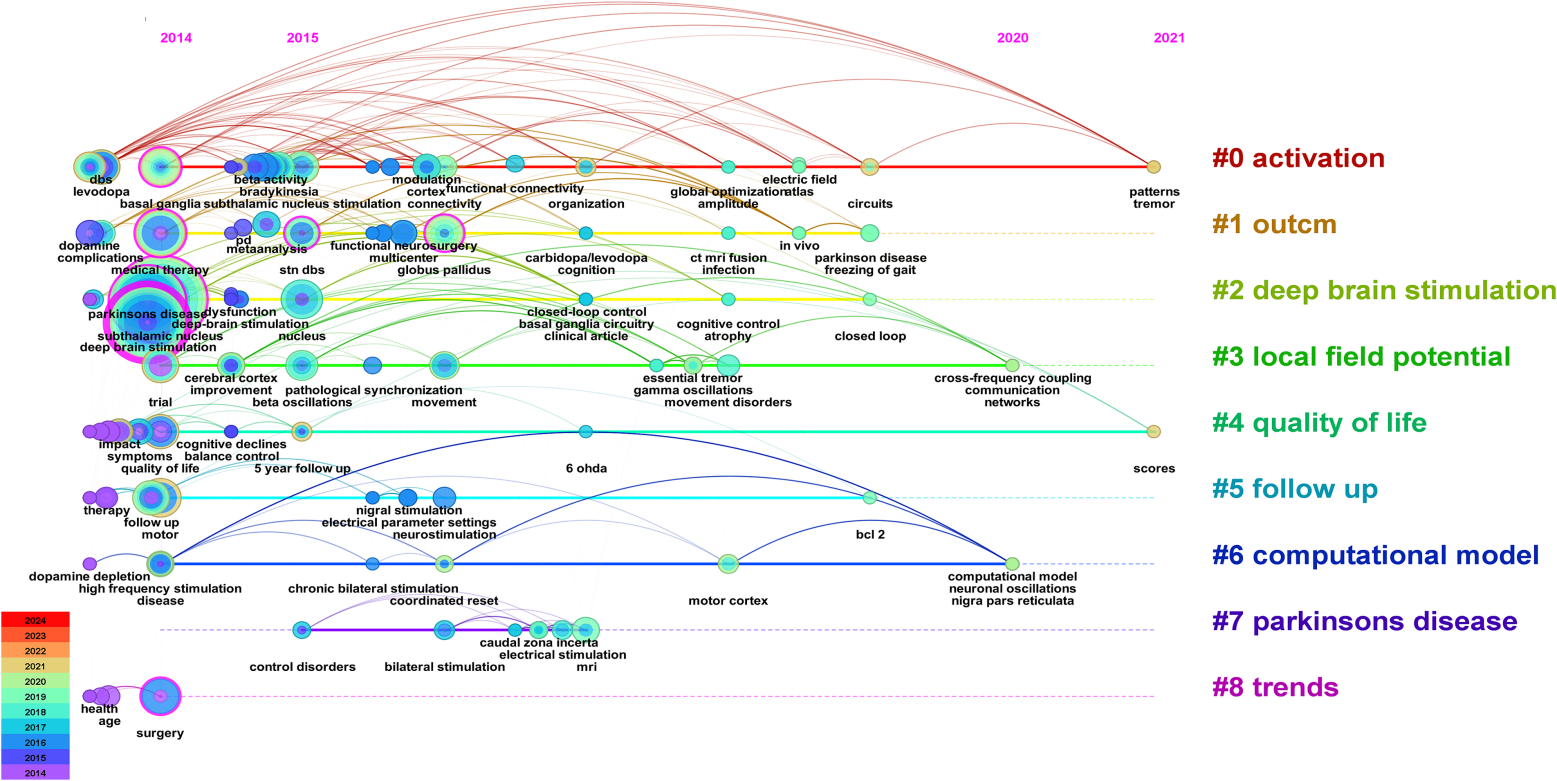
Visual analysis of the 100 most frequently cited papers based on keyword cluster analysis.

**Figure 10:**
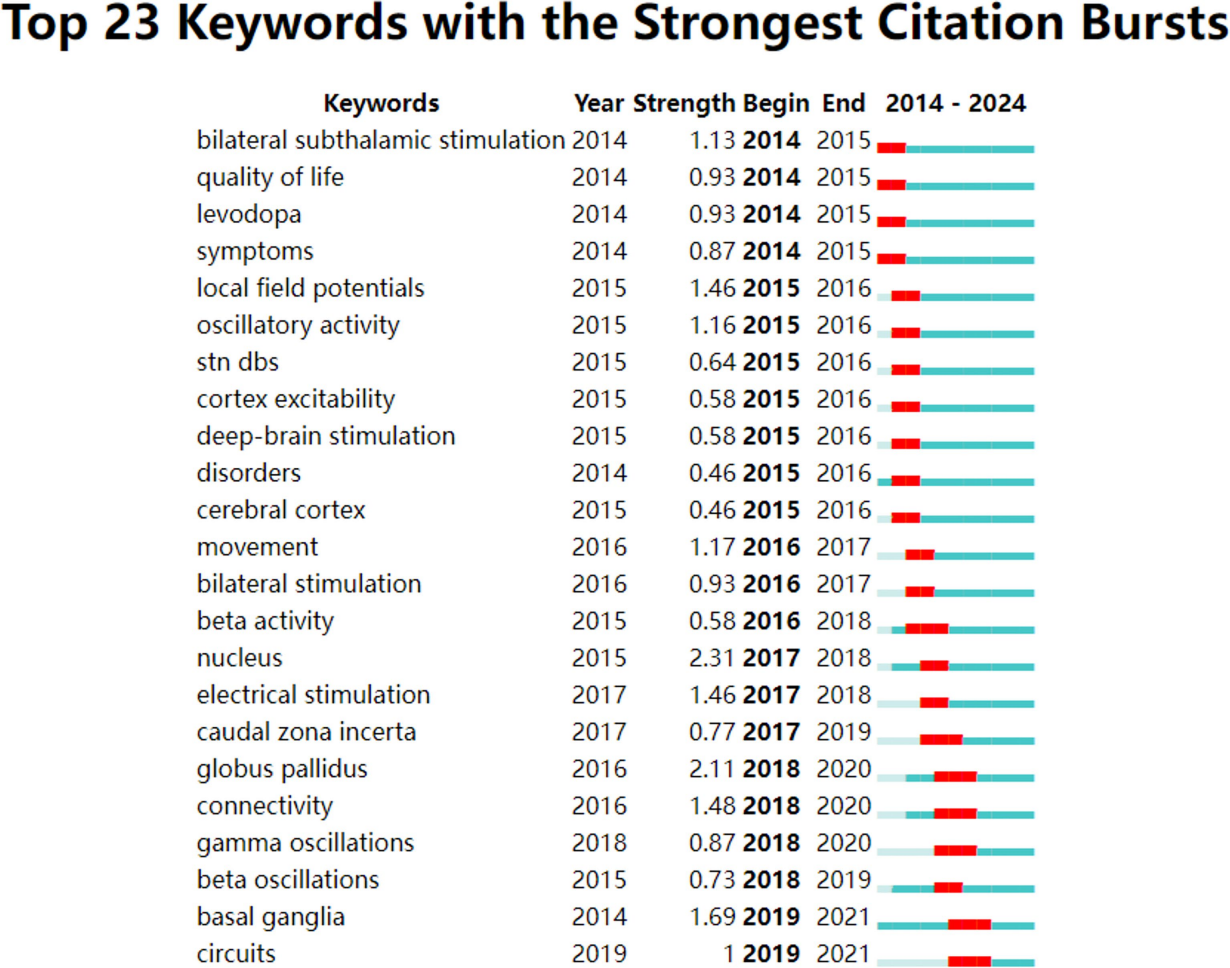
Burst analysis of keywords ranked by citation burst intensity.

Figures 9, 10 illustrate the evolving trends of keywords over time. The volume of research on DBS therapy for PD surged in 2014, coinciding with a substantial increase in targeted studies on stimulation sites, advancements in surgical techniques, and postoperative evaluation of medication efficacy. Assessment of quality of life and activation of degenerative nerves remained pivotal elements throughout the research, with sustained exploration over time. Research on computational models started early but with relatively few related articles. Since 2014, research in this field has steadily progressed with a wide-ranging scope, rapidly updating hotspots, and progressively deeper insights. In recent years, researchers have shifted their focus to studies on “parkinson disease freezing of gait,” “cross-frequency coupling communication networks,” “BG,” “gamma oscillations” and “beta oscillations.” It was evident that with ongoing exploration in neurophysiology and biophysics, research hotspots gradually shifted from conventional treatment approaches to exploring innovative stages. In 2014, the bilateral STN and levodopa were focal points of research, whereas by 2018, gamma oscillations and beta oscillations, among other electrophysiological feedback signals, became mainstream subjects of investigation, leading to the development of related research. For instance, Mengchen Yin et al. reported that the abnormal increase in *β*-*γ* phase-amplitude coupling in the primary motor cortex of PD patients may be associated with the occurrence of freezing during gait (Yin Z. et al., 2022).

#### 3.5.4 Analysis of cocited articles and cited references

**Figure 11:**
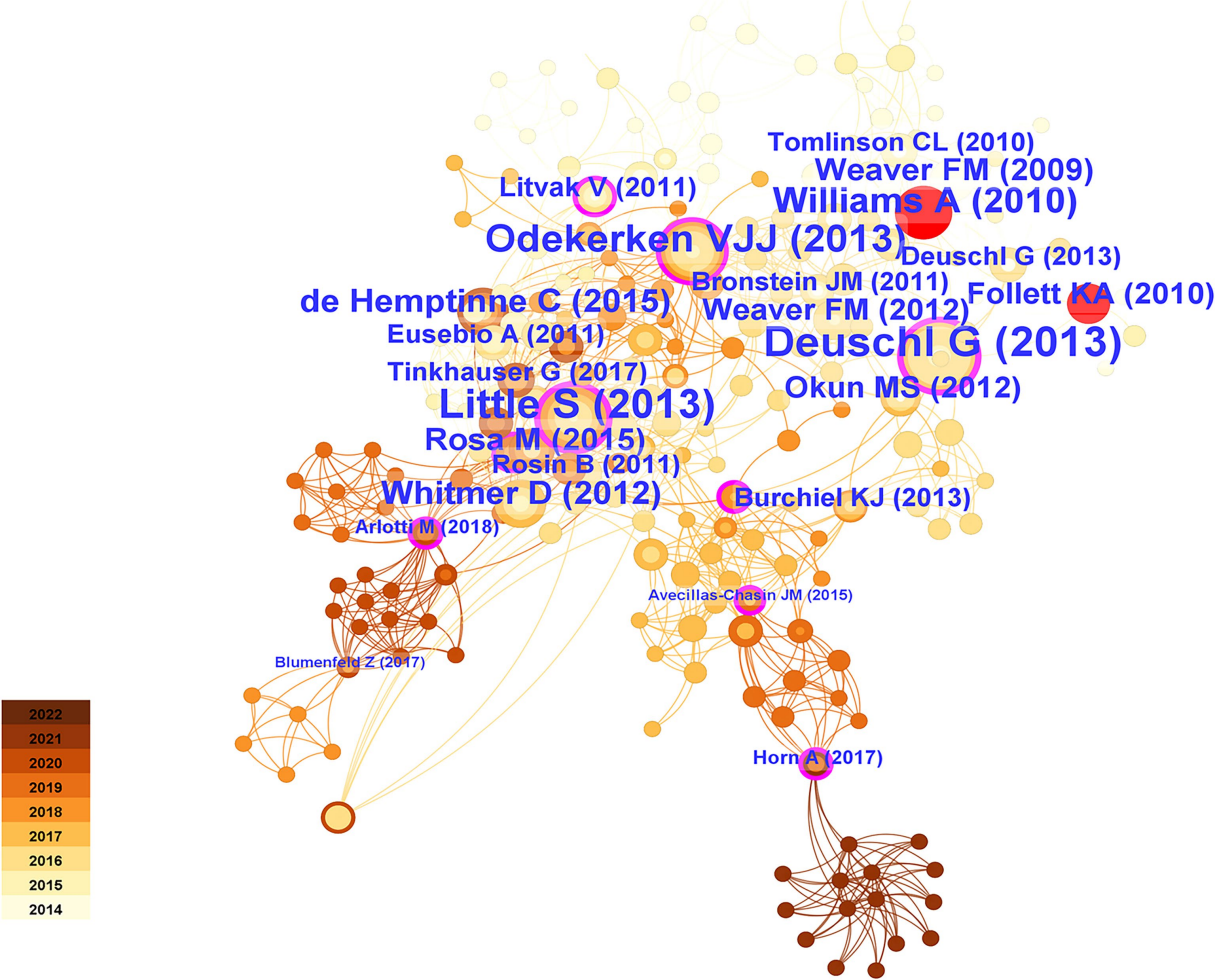
Cocited articles of the 100 most frequently cited papers based on cluster analysis.

Citation relationships refer to situations in which multiple articles were cited by other articles simultaneously. This relationship has been widely used to assess the connections between publications and is an important research parameter. To achieve this goal, we utilized CiteSpace software to analyze cocitation patterns and identify prominent instances of literature being cited. Figure 11 displays the most frequently cited references. Table 3 presented the top 25 most popular articles. Peter Brown is the most frequent corresponding author (Schuepbach et al., 2013) proposed that DBS, compared to medication therapy, was suitable for treating PD patients with early motor complications, expanding the treatment window for DBS beyond late-stage PD (Simon et al., 2016) proposed in 2018 that real-time personalized and optimized adaptive DBS (aDBS) can be achieved through brain-computer interfaces (BCIs) monitoring local field potentials, innovating the monitoring approach for this device (Williams et al., 2010) performed a randomized open-label research to examine self-reported quality of life in 39 Parkinson’s disease patients using the PD Questionnaire (PDQ-39). They found that DBS treatment significantly improved quality of life in patients with advanced PD and that this treatment was beneficial.

**Table 3:** The top 25 most cited references.

DOI	First author	Corresponding author	Journal	Title	Begin	Strength
10.1016/S1474-4422(10)70093-4	Adrian Williams	Natalie Ives	Lancet Neurol	Deep brain stimulation plus best medical therapy versus best medical therapy alone for advanced Parkinson’s disease (PD SURG trial): a randomized, open-label trial	2014	4.91
10.1056/NEJMoa0907083	Kenneth A. Follett	Frances M. Weaver	The new england journal of medicine	Pallidal versus Subthalamic Deep-Brain Stimulation for Parkinson’s Disease	2014	2.83
10.1002/mds.23429	Claire L. Tomlinson	Claire Tomlinson	Movement Disorders	Systematic Review of Levodopa Dose Equivalency Reporting in Parkinson’s Disease	2014	2.01
10.1016/j.neuron.2012.09.032	Qian Li	Ya Ke and Wing-Ho Yung	Neuron	Therapeutic Deep Brain Stimulation in Parkinsonian Rats Directly Influences Motor Cortex	2014	1.6
10.1093/brain/awq332	Vladimir Litvak	Peter Brown	Brain	Resting oscillatory cortico-subthalamic connectivity in patients with Parkinson’s disease	2014	1.01
10.1212/WNL.0b013e31825dcdc1	Frances M. Weaver	Frances M. Weaver	Neurology	Randomized trial of deep brain stimulation for Parkinson disease	2015	2.02
10.1136/innp.2010.217489	A Eusebio	Peter Brown	J Neurol Neurosurg Psychiatry	Deep brain stimulation can suppress pathological synchronization in parkinsonian patients	2015	1.43
10.1016/S1474-4422(12)70264-8	Vincent J J Odekerken	Rob M A de Bie	Lancet Neurol	Subthalamic nucleus versus globus pallidus bilateral deep brain stimulation for advanced Parkinson’s disease (NSTAPS study): a randomized controlled trial	2015	1.23
10.1016/S1474-4422(13)70294-1	Anna Castrioto	Prof Paul Krack	Lancet Neurol	Mood and behavioral effects of subthalamic stimulation in Parkinson’s disease	2015	1.14
10.1016/j.expneurol.2012.09.013	Alberto Priori	Alberto Priori	Experimental Neurology	Adaptive deep brain stimulation (aDBS) controlled by local field potential oscillations	2015	1.14
10.1002/ana.23951	Simon Little	Peter Brown	ANN NEUROL	Adaptive Deep Brain Stimulation in Advanced Parkinson Disease	2016	1.58
10.1002/acn3.168	Martin M	Jens Volkmann	Annals of Clinical and Translational Neurology	Short pulse width widens the therapeutic window of subthalamic neurostimulation	2016	1.27
10.1093/brain/awu027	Joshua Kahan	Tom Foltynie	Brain	Resting state functional MRI in Parkinson’s disease: the impact of deep brain stimulation on ‘effective’ connectivity	2016	1.27
10.3171/2013.4JNS122324	Kim J. Burchiel	Ahmed M. Raslan	J Neurosurg	Accuracy of deep brain stimulation electrode placement using intraoperative computed tomography without microelectrode recording	2016	1.07
10.1136/innp-2013-30607	Iciar Aviles-Olmos	Patricia Limousin	J Neurol Neurosurg Psychiatry	Long-term outcome of subthalamic nucleus deep brain stimulation for Parkinson’s disease using an MRI-guided and MRI-verified approach	2017	1.45
10.1093/brain/aww182	Ettore A. Accolla	Ettore Accolla	Brain	Brain networks modulated by subthalamic nucleus deep brain stimulation	2017	1.45
10.1136/innp-2015-310972	Simon Little	Peter Brown	Movement disorders	Bilateral adaptive deep brain stimulation is effective in Parkinson’s disease	2018	2.02
10.1136/jnnp-2016-313518	Simon Little	Peter Brown	J Neurol Neurosurg Psychiatry	Adaptive deep brain stimulation for Parkinson’s disease demonstrates reduced speech side effects compared to conventional stimulation in the acute setting	2018	2.02
10.1038/nn.3997	Coralie de Hemptinne	Coralie de Hemptinne	nature NEUROSCIENCE	Therapeutic deep brain stimulation reduces cortical phase-amplitude coupling in Parkinson’s disease	2018	1.96
10.1002/mds.26241	Manuela Rosa	Manuela Rosa	Movement Disorders	Adaptive Deep Brain Stimulation in a Freely Moving Parkinsonian Patient	2018	1.95
10.1016/i.neuroimage.2017.07.012	Harith Akram	Harith Akram	NeuroImage	Subthalamic deep brain stimulation sweet spots and hyperdirect cortical connectivity in Parkinson’s disease	2018	1.51
10.1088/1741-2552/aabc9b	Nicole C Swann	Philip A. Starr	Journal of Neural Engineering	Adaptive deep brain stimulation for Parkinson’s disease using motor cortex sensing	2019	2.21
10.1212/WNL.0000000000005121	Mattia Arlotti	Alberto Priori	American Academy of Neurology	Eight-hours adaptive deep brain stimulation in patients with Parkinson disease	2019	1.65
10.1002/ana.24974	Andreas Horn	Andreas Horn	Annals of Neurology	Connectivity predicts deep brain stimulation outcome in Parkinson’s disease	2019	1.51
10.1016/i.nbd.2018.09.004	Chioma M. Anidi	Helen M. Bronte-Stewart	Neurobiology of Disease	Neuromodulation targets pathological not physiological beta bursts during gait in Parkinson’s disease	2019	1.1

According to the time of emergence, three time periods could be distinguished: 2014–2015, 2016–2017, and 2018–2019. During the first time period, the main research focus was the comparative effectiveness of DBS targets in treatment. The STN was more suitable than the globus pallidus internus (GPi) for treating patients with dementia symptoms (Frances et al., 2012) and reducing the need for dopaminergic medication. However, STN was more suitable for improving the severity of depression (Kenneth et al., 2010). During the second period, research primarily focused on the connectivity between brain regions and the validation of functional MRI guidance. The medial temporal lobe (including the amygdala and hippocampus) is mainly connected to the ventral subthalamic area (Accolla et al., 2016). STN-DBS regulates all major components of the motor cortex-striatum-thalamus-cortex loop, with stimulation decreasing the strength of both incoming and outgoing signals to the STN, thereby enhancing the cortical-striatal, thalamocortical, and direct pathways (Kahan et al., 2014). Functional magnetic resonance imaging (fMRI) is a valuable technique for detecting and validating the aforementioned findings (Burchiel et al., 2013). During the third time period, increased attention was given to the functional aspects of brain regions, alongside the emergence of aDBS. The functional status of the prefrontal cortex is particularly relevant to the stiffness symptoms experienced by patients (Akram et al., 2017). aDBS not only significantly conserved energy (38–45%) but also demonstrated good therapeutic efficacy (Swann et al., 2018). It could improve axial and limb symptoms and track stimulation needs across different medication states (Little et al., 2016) and provide a wider therapeutic window for impaired speech clarity (Little Simon et al., 2016).

## 4 Discussion

We investigated the top 100 most frequently cited publications using bibliometric and visualization analytic approaches, with the goal of exploring research hotspots and worldwide trends in DBS treatment for Parkinson’s disease (Liang et al., 2020). By leveraging specific and reliable metrics, bibliometric analysis enables the characterization of publications (Andrade-Maia et al., 2023; Huang et al., 2024; Zhou et al., 2023). Assessing the most frequently cited publications in the medical sector can give useful insights for researchers in understanding the existing environment and guiding the development of future research initiatives (Cheng et al., 2022; Ding et al., 2022; Yin M. et al., 2022).

Among the top 100 most referenced papers, one-third could be traced back to the top 15. Horn and De Hemptinne primarily investigated the improvement of Parkinsonian motor symptoms through DBS implants based on brain region connectivity networks. Citation analysis revealed that their research garnered the most citations (de Hemptinne et al., 2015; Horn et al., 2017), suggesting that future research on DBS implantation based on brain region connectivity networks may be a breakthrough in improving PD symptoms.

Based on co-occurrence analysis and keyword burst analysis using CiteSpace and VOSviewer, we found that in recent years, keywords describing STN, DBS, PD, medical therapy, BG, and STN stimulation have the highest frequency of usage. There has been a focus on LFP detection in brain regions, the development of computer models, the assessment of therapeutic outcomes, and the investigation of quality of life. We should also pay attention to biomarkers, intraoperative lead placement strategies, postoperative programming protocols, and animal experimental research used to explore this field. As research on DBS for the treatment of PD continues to deepen, researchers are increasingly focusing on biomarkers. Modulation of beta oscillations and their phase-amplitude interaction with gamma oscillations are thought to convey pathogenic information regarding motor diseases (Bouthour et al., 2019). Uric acid, as a biomarker for PD, could be utilized to predict the therapeutic outcomes of STN-DBS (Chang et al., 2023). We should explore biomarkers more extensively, as they can aid in predicting disease progression, guiding drug development, and facilitating personalized medicine. The precision of intraoperative lead placement is directly related to the improvement rate of postoperative symptoms. Ostrem et al. (2016) reported that the use of the ClearPoint interventional real-time MRI-guided method for DBS lead placement can achieve highly accurate lead placement. The median MCP was not as effective as the medial border of the STN in serving as an anatomical location associated with motor improvement, and it was also not as precise as identifying the optimal position within the nucleus (Bot et al., 2018). Improving the precision of intracranial electrode implantation is of concern and warrants further research. Programmer scientists are continuously exploring single/double stimulation types of electrodes as well as aDBS or closed loop DBS (Fleming et al., 2020; Little et al., 2016; Swann et al., 2018; Tinkhauser et al., 2017). Future efforts should also concentrate on how to minimize complications following surgery (Olson et al., 2023). Accurate delivery of the appropriate stimulation dose to the target area is key to successful DBS. Boutet et al. (2021) combined fMRI with machine learning to propose that the generation of optimal stimulation in clinical practice was characterized by an fMRI brain response pattern that preferentially engaged motor circuits. This offered the possibility of deriving optimal stimulation for fMRI-guided DBS programming. Postoperative programming is essential for ensuring the efficacy of DBS. Rapidly identifying accurate stimulation parameters helps to reduce the occurrence of stimulation-related complications. There is a need for scientists to better explore stimulation protocols. Some researchers have already begun testing new stimulation techniques on animals (Wang et al., 2016; Xiao et al., 2019).

Through keyword clustering analysis, we found that the keywords are categorized into the following groups: #0, activation; #1, outcome; #2, deep brain stimulation; #3, local field potential; #4, quality of life; #5, follow-up; #6, computational model; #7, Parkinson’s disease; and #8, trends. As the condition progresses, the therapeutic effects of medication on PD symptoms might gradually diminish, leading to symptom fluctuations and dyskinesias, which directly impact the quality of life of patients. DBS could serve as an adjunct to medication therapy to improve this situation, thereby enhancing the quality of life for patients (Williams et al., 2010). There was a close relationship between #0 activation, #5 follow-up, #2 deep brain stimulation, and #3 local field potentials. The efficacy of DBS treatment was closely associated with the activation of the target area. For instance, local field potential (LFP) activity serves as a crucial feedback signal in the DBS system, leading to numerous clinical trials. Additionally, postoperative follow-up and retrospective analysis are essential methods for tracking the therapeutic effects of treatment, providing critical data for evaluating the therapeutic potential of DBS in treating diseases such as PD. Additionally, trends in quality of life in quadrants #4, #1, #5, and #8 exhibited high coherence, indicating a growing trend toward postoperative quality of life follow-up, which could assist in optimizing treatment outcomes. The close relationships between #0 activation, #2 deep brain stimulation, #3 local field potentials, and the #6 computational model suggested that computational modeling was an emerging auxiliary tool based on recording LFP activity. By constructing models of neurophysiological changes during DBS and exploring the dynamic behavior of the Parkinson’s network, this study could provide inspiration and insights for further investigation into DBS therapy for PD.

Additionally, the mechanism by which DBS treats PD remains unclear and has been a focal point of interest for researchers. The benefits of DBS for PD patients are contingent upon the connections between the stimulated sites and various brain regions, and the relationships among these areas and connections are equally awaiting our investigation. Below, we provided a complete assessment and discussion of the present status of research, hot themes, and future trends in two areas: therapeutic targets and DBS treatment methods for Parkinson’s disease.

### 4.1 Mechanism of action

DBS is an efficacious therapeutic method that modulates aberrant neural activity by implanting electrodes in specific brain regions and providing electrical stimulation through an external device. Although the mechanism underlying PD is not fully understood, researchers hold varying perspectives on the mechanism by which DBS treats PD. Initially, DBS was believed to alleviate motor symptoms of PD by inhibiting overactive neurons in the basal ganglia. However, as research progresses, it has become evident that DBS may function through complex neural network regulatory mechanisms. Current research primarily focuses on the analysis of neuro-oscillation phase and amplitude coupling, as well as the understanding of the cortico-basal-ganglia loop. A study documented DBS electrodes in PD patients during simultaneous stimulation of the same target (pulse width 60 μs; frequency 130 Hz) and revealed that DBS could suppress pathological 11–30 Hz activity near the stimulation site in PD patients. This indicates that STN-DBS may improve bradykinesia and rigidity by inhibiting *β*-frequency activity in the STN area (Eusebio et al., 2011). In an experiment conducted by Li et al. (2012), it was discovered that random reverse spikes originating from the STN directly altered the firing probability of corticofugal projection neurons in the cortex, breaking the dominant role of the β rhythm and thereby reestablishing control over the subject’s motor function. This research implies that DBS treatment for Parkinson’s disease patients improves motor function via modifying the firing probability of corticofugal projection neurons in the cerebral cortex. The analysis of the neural oscillation phase and amplitude coupling, as important mechanisms of large-scale brain network dynamics, has garnered widespread attention. de Hemptinne et al. (2015) proposed that DBS of the basal ganglia improves the cortex in patients with PD by reducing the excessive beta phase locking of motor cortical neurons. Acute therapeutic DBS may improve the motor signs of PD by reversibly decreasing phase-amplitude interactions. DBS might alleviate symptoms by eliminating the relative, time-specific increase in STN beta oscillations (Herz et al., 2018), or it might improve motor symptoms by potentially increasing gamma power (Muthuraman et al., 2020). Based on frequency-specific optogenetic findings, STN-DBS indeed improves motor symptoms in PD patients by reducing abnormal oscillatory activity within STN-related neural circuits (Yu et al., 2020). Since the discovery in 2014 that STN-DBS modulates the motor cortex-striatum-thalamus-cortex loop (Kahan et al., 2014), its impact on the synchronized activity of the cortico-basal ganglia loop still remains to be fully characterized. They (Oswal et al., 2016) suggested that STN-DBS can affect the coupling mediated by the hyperdirect pathway of the STN, thereby suppressing the connection between the cortex and the thalamostriatal complex in the high-beta frequency band. Moreover, the clinical efficacy of DBS parameters intensifies this effect (Spooner et al., 2024). As computer modeling technology advances, the field of research on the therapeutic mechanisms of DBS for PD is becoming increasingly intelligent and precise, offering researchers more convenient avenues for exploration. This (Saenger et al., 2017) was the first to apply computers to DBS, with computational models providing direct information. We utilized computational connectomics to simulate the impact of therapeutic DBS on overall brain activity in PD patients and found that the treatment most likely affected areas such as the right thalamus, GP, and orbitofrontal cortex. By recording the intracranial LFP of STN-DBS implants during a visual attention task, steady-state visual evoked potentials (SSVEPs) were derived, and a causal relationship was established. It was ultimately discovered that the suppressive modulation of attention in PD patients by STN-DBS may be mediated through the inhibition of auditory-induced suppression of SSVEPs (Soh et al., 2024). It is important to recognize that due to the unclear pathophysiology of PD, the study of the therapeutic mechanisms of DBS has also undergone intensive exploration. Researchers are proposing various possibilities to explain the therapeutic effects of DBS on PD. Although this process is full of challenges, it also promotes a better understanding of the disease mechanisms of PD among researchers (Brown and De Jesus, 2024).

### 4.2 Therapeutic target

Currently, DBS is primarily used to treat the major motor symptoms of Parkinson’s disease. Among the 100 most frequently referenced studies, STN and GPi were the major targets. More than half of these papers discuss the application of the STN in PD patients. STN has been proven to have considerable therapeutic benefits on the primary motor symptoms of Parkinson’s disease, such as bradykinesia, muscular stiffness, and limb tremors, and it can also significantly reduce the need for dopaminergic drugs after surgery. DBS significantly improves the quality of life for patients with PD, but its effects tend to diminish over time. A follow-up study revealed that among patients who underwent STN-DBS treatment, the greatest improvement in quality of life occurred within the first three years, with a slight decrease in therapeutic efficacy after five years (Jiang et al., 2015). STN-DBS may also ameliorate nonmotor symptoms such as sleep issues, anxiety, and pain, but it can potentially lead to postoperative cognitive decline. In addition to the STN, the GPi can effectively alleviate the primary motor symptoms of PD. However, compared to GPi-DBS, STN-DBS shows superior efficacy in improving motor symptoms, while GPi-DBS is more beneficial for postoperative cognitive function preservation in patients. Therefore, for patients with cognitive decline or mood disorders, GPi-DBS is preferentially considered (Lachenmayer et al., 2021). For elderly patients presenting with cognitive deficits and a greater number of psychiatric comorbidities, the recommendation is to opt for GPi-DBS. According to recent reports, researchers have gradually shifted their focus toward the exploration of novel therapeutic targets. There are various perspectives among scholars regarding the selection of targets for the treatment of tremor symptoms in patients with PD. Rubens Gisbert Cury et al. reported that the ventral intermediate nucleus of the thalamic ventral intermediate nucleus (ViM) has long-term (over 10 years) effective control of tremor, but its ability to improve other motor symptoms is limited, and it does not ameliorate motor complications. Therefore, ViM-DBS is suitable for PD patients whose primary or sole symptom is tremor (Cury et al., 2017). Researchers have discovered that the dentato-rubro-thalamic tract located in the posterior subthalamic area shows good improvement in idiopathic Parkinson’s syndrome, which is tremor dominant and equivalent in type, making it suitable for treating patients with tremor as the primary symptom. Moreover, it has fewer side effects than ViM-DBS (Coenen et al., 2016). Stimulation of the caudal zona incerta (cZi) also had positive effects on Parkinsonian tremor (Blomstedt et al., 2018). For postural instability and balance problems, the pedunculopontine nucleus (PPN) is a prospective target. According to research, the PPN-DBS plays a significant role in regulating freezing of gait and loss of postural control (Baumgartner et al., 2022). Placing the electrode contacts at the level of the caudal mesencephalic reticular formation (cMRF), which includes the posterior pedunculopontine nucleus and the cuneiform nucleus within the pontomesencephalic junction, is an excellent choice (Goetz et al., 2019), despite some conflicting findings in the literature (Bourilhon et al., 2022). For the treatment of nonmotor symptoms, the nucleus basalis of Meynert (NBM) has shown positive effects. The NBM is a sensitive marker for cholinergic activity. Reports suggest that intermittent NBM, rather than continuous low-frequency stimulation, may improve cognitive function (Sasikumar et al., 2023). However, a randomized controlled trial comprising 117 PD patients with cognitive decline examined the cognitive function of patients under DBS-off and DBS-on circumstances over a six-week period and found no significant difference in cognitive outcomes between the two states (Gratwicke et al., 2018). The orbitofrontal cortex region can provide significant assistance in the search for new stimulation targets (Saenger et al., 2017).

Since the US FDA’s approval of DBS for the treatment of PD in 1997, DBS has become one of the standard options for PD treatment. However, despite its notable efficacy, there are ongoing challenges in resource allocation. Issues such as the difficulty of follow-up care, the high costs associated with the procedure, and the limited accessibility for individuals with lower incomes continue to exist (Willis et al., 2014). These challenges contribute to an uneven distribution of resources. Although the number of patients undergoing DBS surgery for PD is gradually increasing, it is still not sufficient to cover all potentially eligible patients. In particular, patients in low- and middle-income countries lack guaranteed access to treatment. The issue of uneven coverage and uncoordinated development needs to be addressed. Therefore, there is a need for increased focus on case monitoring in the future to ensure the rational allocation of resources. Researchers should engage in more international cooperation, as collaboration between nations is crucial for the research and development of DBS treatment for PD and for understanding its evolving patterns. This can facilitate the coordinated and balanced development of DBS treatment across society.

## 5 Limitations

This is the first bibliometric analysis of the literature on DBS treatment for PD, and it should be noted that the study has significant limitations. Despite the WoSCC is one of the most commonly used academic search databases, it does not index all publications. To ensure the accuracy of our analysis, we utilized title keyword searches rather than subject keyword searches. While our search results have a certain level of precision, there are still limitations. Including only English-language articles may introduce bias in the selection process. Because we focused on the top 100 most referenced publications in the subject over the last decade, more recently released items tend to be disadvantaged. Consequently, all the articles obtained were from the last seven years, which presents a certain limitation. This also indicates that the field has been in an intensive research phase in recent years, and researchers are advised to continue their efforts in exploring relevant technologies and their applications. Due to data and methodological limitations, this study was unable to assess the specific impact of senior author migration on institutional research output. It is suggested that future research could explore it for a more comprehensive analytical perspective.

## 6 Conclusion

To our knowledge, this is the first bibliometric review of the most frequently referenced papers on DBS therapy for Parkinson’s disease. Our research indicates that the most significant research on DBS has been published in the journal Neurology. Marwan Hariz has published the most articles. The UCL is the most prolific institution. The field has seen steady progress in technological exploration, target identification, and investigation of therapeutic mechanisms. In recent years more research has been done on activation, outcome, local field potential, quality of life, follow-up, computational model, and trends. In the field of DBS for PD, countries with large economies such as the US, UK, and Germany have a clear position in the field of DBS for PD. Going forward, we believe that research could focus on uncovering mechanisms of action, discovering biomarkers, refining therapeutic targets, and developing precise localization techniques to prolong treatment outcomes and improve quality of life postoperatively, leading to personalized, cost-effective treatment plans and balanced and coordinated development among countries.

## Footnotes

^1^http://www.R-project.org

^2^https://data.worldbank.org/
