# Supplementary material for: Deep brain stimulation for Parkinson’s disease: bibliometric analysis of the top 100 cited literature
Source: Front Aging Neurosci. 2024 Oct 16;16:1413074. doi: 10.3389/fnagi.2024.1413074 (PMC11521828; doi:10.3389/fnagi.2024.1413074)
Supplement: Supplementary file 1 [file Table_1.docx]

Supplementary Table 1: DBS’s top 100 most cited papers until 2024.

| Rank | Title | Author | Year | Journal | Total citation | Average citation by year | References |
| --- | --- | --- | --- | --- | --- | --- | --- |
| 1 | Therapeutic deep brain stimulation reduces cortical phase-amplitude coupling in Parkinson's disease | DE HEMPTINNE C | 2015 | NATURE NEUROSCIENCE | 400 | 44.44 | 56 |
| 2 | Connectivity predicts Deep Brain Stimulation outcome in Parkinson disease | HORN A | 2017 | ANNALS OF NEUROLOGY | 394 | 56.29 | 51 |
| 3 | The modulatory effect of adaptive deep brain stimulation on beta bursts in Parkinson's disease | TINKHAUSER G | 2017 | BRAIN | 250 | 35.71 | 29 |
| 4 | Adaptive deep brain stimulation for Parkinson's disease using motor cortex sensing | SWANN NC | 2018 | JOURNAL OF NEURAL ENGINEERING | 225 | 37.50 | 27 |
| 5 | Bilateral adaptive deep brain stimulation is effective in Parkinson's disease | LITTLE S | 2016 | JOURNAL OF NEUROLOGY NEUROSURGER-Y AND PSYCHIATRY | 215 | 26.88 | 27 |
| 6 | GPi vs STN deep brain stimulation for Parkinson disease three-year follow-up | ODEKERKEN VJJ | 2016 | NEUROLOGY | 166 | 20.75 | 37 |
| 7 | Beta oscillations in freely moving Parkinson's subjects are attenuated during deep brain stimulation | QUINN EJ | 2015 | MOVEMENT DISORDERS | 165 | 18.33 | 19 |
| 8 | Deep brain stimulation modulates synchrony within spatially and spectrally distinct resting state networks in Parkinson's disease | OSWAL A | 2016 | BRAIN | 163 | 20.38 | 77 |
| 9 | Resting state functional mri in Parkinson's disease: the impact of deep brain stimulation on 'effective' connectivity | KAHAN J | 2014 | BRAIN | 163 | 16.30 | 83 |
| 10 | Thalamic deep brain stimulation for tremor in Parkinson disease, essential tremor, and dystonia | CURY RG | 2017 | NEUROLOGY | 156 | 22.29 | 40 |
| 11 | Subthalamic deep brain stimulation sweet spots and hyperdirect cortical connectivity in Parkinson's disease | AKRAM H | 2017 | NEUROIMAGE | 149 | 21.29 | 106 |

Table 2 (Continued)

| Rank | Title | Author | Year | Journal | Total citation | Average citation by year | References |
| --- | --- | --- | --- | --- | --- | --- | --- |
| 12 | Eight-hours adaptive deep brain stimulation in patients with Parkinson disease | ARLOTTI M | 2018 | NEUROLOGY | 145 | 24.17 | 9 |
| 13 | Dual threshold neural closed loop deep brain stimulation in Parkinson disease patients | VELISAR A | 2019 | BRAIN STIMULATION | 138 | 27.60 | 26 |
| 14 | Programming deep brain stimulation for Parkinson's disease: the toronto western hospital algorithms | PICILLO M | 2016 | BRAIN STIMULATION | 137 | 17.13 | 107 |
| 15 | Long-term outcome of subthalamic nucleus deep brain stimulation for Parkinson's disease using an mri-guided and mri-verified approach | AVILES-OLMOS I | 2014 | JOURNAL OF NEUROLOGY NEUROSURGERY AND PSYCHIATRY | 134 | 13.40 | 38 |
| 16 | Multiple-source current steering in subthalamic nucleus deep brain stimulation for Parkinson's disease (the vantage study): a non-randomised, prospective, multicentre, open-label study | TIMMERMANN L | 2015 | LANCET NEUROLOGY | 119 | 13.22 | 27 |
| 17 | Clinical outcomes using clearpoint interventional mri for deep brain stimulation lead placement in Parkinson's disease | OSTREM JL | 2016 | JOURNAL OF NEUROSURGERY | 109 | 13.63 | 34 |
| 18 | Probabilistic sweet spots predict motor outcome for deep brain stimulation in Parkinson disease | DEMBEK TA | 2019 | ANNALS OF NEUROLOGY | 102 | 20.40 | 49 |
| 19 | Biomarkers for closed-loop deep brain stimulation in Parkinson disease and beyond | BOUTHOUR W | 2019 | NATURE REVIEWS NEUROLOGY | 99 | 19.80 | 142 |
| 20 | Subthalamic nucleus deep brain stimulation in early stage Parkinson's disease | CHARLES D | 2014 | ParkinsonISM & RELATED DISORDERS | 99 | 9.90 | 22 |
| 21 | Adaptive deep brain stimulation in Parkinson's disease | BEUDEL M | 2016 | ParkinsonISM & RELATED DISORDERS | 91 | 11.38 | 28 |
| 22 | Effects of deep brain stimulation on pain and other nonmotor symptoms in Parkinson disease | CURY RG | 2014 | NEUROLOGY | 91 | 9.10 | 40 |

Table 2 (Continued)

| Rank | Title | Author | Year | Journal | Total citation | Average citation by year | References |
| --- | --- | --- | --- | --- | --- | --- | --- |
| 23 | Predicting optimal deep brain stimulation parameters for Parkinson's disease using functional mri and machine learning | BOUTET A | 2021 | NATURE COMMUNICATIONS | 90 | 30.00 | 62 |
| 24 | Effects of deep brain stimulation of the subthalamic nucleus on freezing of gait in Parkinson's disease: a prospective controlled study | VERCRUYSSE S | 2014 | JOURNAL OF NEUROLOGY NEUROSURGERY AND PSYCHIATRY | 90 | 9.00 | 40 |
| 25 | Disparities in deep brain stimulation surgery among insured elders with Parkinson disease | WILLIS AW | 2014 | NEUROLOGY | 89 | 8.90 | 27 |
| 26 | Bilateral deep brain stimulation of the nucleus basalis of meynert for Parkinson disease dementia a randomized clinical trial | GRATWICKE J | 2018 | JAMA NEUROLOGY | 88 | 14.67 | 43 |
| 27 | Disparities in access to deep brain stimulation surgery for Parkinson disease interaction between african american race and medicaid use | CHAN AK | 2014 | JAMA NEUROLOGY | 87 | 8.70 | 49 |
| 28 | Neuropsychological changes following deep brain stimulation surgery for Parkinson's disease: comparisons of treatment at pallidal and subthalamic targets versus best medical therapy | ROTHLIND JC | 2015 | JOURNAL OF NEUROLOGY NEUROSURGERY AND PSYCHIATRY | 85 | 9.44 | 21 |
| 29 | Coordinated reset deep brain stimulation of subthalamic nucleus produces long-lasting, dose-dependent motor improvements in the 1-methyl-4-phenyl-1,2,3,6-tetrahydropyridine non-human primate model of Parkinsonism | WANG J | 2016 | BRAIN STIMULATION | 83 | 10.38 | 70 |
| 30 | Closed-loop deep brain stimulation effects on Parkinsonian motor symptoms in a non-human primate - is beta enough? | JOHNSON LA | 2016 | BRAIN STIMULATION | 79 | 9.88 | 30 |

Table 2 (Continued)

| Rank | Title | Author | Year | Journal | Total citation | Average citation by year | References |
| --- | --- | --- | --- | --- | --- | --- | --- |
| 31 | Deep brain stimulation induced normalization of the human functional connectome in Parkinson's disease | HORN A | 2019 | BRAIN | 78 | 15.60 | 83 |
| 32 | Pallidal deep-brain stimulation disrupts pallidal beta oscillations and coherence with primary motor cortex in Parkinson's disease | WANG DD | 2018 | JOURNAL OF NEUROSCIENCE | 78 | 13.00 | 62 |
| 33 | Deep brain stimulation: a paradigm shifting approach to treat Parkinson's disease | HICKEY P | 2016 | FRONTIERS IN NEUROSCIENCE | 78 | 9.75 | 88 |
| 34 | Deep-brain stimulation associates with improved microvascular integrity in the subthalamic nucleus in Parkinson's disease | PIENAAR IS | 2015 | NEUROBIOLOGY OF DISEASE | 76 | 8.44 | 74 |
| 35 | Awake versus asleep deep brain stimulation for Parkinson's disease: a critical comparison and meta-analysis | HO AL | 2018 | JOURNAL OF NEUROLOGY NEUROSURGERY AND PSYCHIATRY | 75 | 12.50 | 36 |
| 36 | Clinical outcomes of asleep vs awake deep brain stimulation for Parkinson disease | BRODSKY MA | 2017 | NEUROLOGY | 75 | 10.71 | 22 |
| 37 | Neural plasticity in human brain connectivity: the effects of long term deep brain stimulation of the subthalamic nucleus in Parkinson's disease | VAN HARTEVELT TJ | 2014 | PLOS ONE | 75 | 7.50 | 58 |
| 38 | Distinct phenotypes of speech and voice disorders in Parkinson's disease after subthalamic nucleus deep brain stimulation | TSUBOI T | 2015 | JOURNAL OF NEUROLOGY NEUROSURGERY AND PSYCHIATRY | 70 | 7.78 | 40 |
| 39 | Low-frequency versus high-frequency subthalamic nucleus deep brain stimulation on postural control and gait in Parkinson's disease: a quantitative study | VALLABHAJOSULA S | 2015 | BRAIN STIMULATION | 69 | 7.67 | 40 |
| 40 | Clinical outcomes following awake and asleep deep brain stimulation for Parkinson disease | CHEN T | 2019 | JOURNAL OF NEUROSURGERY | 68 | 13.60 | 35 |

Table 2 (Continued)

| Rank | Title | Author | Year | Journal | Total citation | Average citation by year | References |
| --- | --- | --- | --- | --- | --- | --- | --- |
| 41 | Effect of advancing age on outcomes of deep brain stimulation for Parkinson disease | DELONG MR | 2014 | JAMA NEUROLOGY | 67 | 6.70 | 32 |
| 42 | Subthalamic nucleus deep brain stimulation with a multiple independent constant current-controlled device in Parkinson's disease (intrepid): a multicentre, double-blind, randomised, sham-controlled study | VITEK JL | 2020 | LANCET NEUROLOGY | 66 | 16.50 | 22 |
| 43 | Impulse control behaviours in patients with Parkinson's disease after subthalamic deep brain stimulation: de novo cases and 3-year follow-up | AMAMI P | 2015 | JOURNAL OF NEUROLOGY NEUROSURGERY AND PSYCHIATRY | 66 | 7.33 | 17 |
| 44 | The cost-effectiveness of deep brain stimulation in combination with best medical therapy, versus best medical therapy alone, in advanced Parkinson's disease | EGGINGTON S | 2014 | JOURNAL OF NEUROLOGY | 66 | 6.60 | 72 |
| 45 | Probabilistic versus deterministic tractography for delineation of the cortico-subthalamic hyperdirect pathway in patients with Parkinson disease selected for deep brain stimulation | PETERSEN MV | 2017 | JOURNAL OF NEUROSURGERY | 64 | 9.14 | 53 |
| 46 | Differential effects of deep brain stimulation target on motor subtypes in Parkinson's disease | KATZ M | 2015 | ANNALS OF NEUROLOGY | 64 | 7.11 | 36 |
| 47 | Deep brain stimulation in early-stage Parkinson disease five-year outcomes | HACKER ML | 2020 | NEUROLOGY | 63 | 15.75 | 30 |
| 48 | Parkinson's disease outcomes after intraoperative ct-guided "asleep" deep brain stimulation in the globus pallidus internus | MIRZADEH Z | 2016 | JOURNAL OF NEUROSURGERY | 63 | 7.88 | 35 |
| 49 | Quality of life predicts outcome of deep brain stimulation in early Parkinson disease | SCHUEPBACH WMM | 2019 | NEUROLOGY | 62 | 12.40 | 29 |
| 50 | Uncovering the underlying mechanisms and whole-brain dynamics of deep brain stimulation for Parkinson's disease | SAENGER VM | 2017 | SCIENTIFIC REPORTS | 62 | 8.86 | 79 |

Table 2 (Continued)

| Rank | Title | Author | Year | Journal | Total citation | Average citation by year | References |
| --- | --- | --- | --- | --- | --- | --- | --- |
| 51 | An 8-year follow-up on the effect of subthalamic nucleus deep brain stimulation on pain in Parkinson disease | JUNG YJ | 2015 | JAMA NEUROLOGY | 62 | 6.89 | 35 |
| 52 | Spinal cord stimulation improves gait in patients with Parkinson's disease previously treated with deep brain stimulation | DE SOUZA CP | 2017 | MOVEMENT DISORDERS | 61 | 8.71 | 13 |
| 53 | Phasic burst stimulation: a closed-loop approach to tuning deep brain stimulation parameters for Parkinson's disease | HOLT AB | 2016 | PLOS COMPUTATIONAL BIOLOGY | 61 | 7.63 | 52 |
| 54 | Neuropsychological outcome after deep brain stimulation for Parkinson disease | ODEKERKEN VJJ | 2015 | NEUROLOGY | 61 | 6.78 | 28 |
| 55 | Deep brain stimulation improves survival in severe Parkinson's disease | NGOGA D | 2014 | JOURNAL OF NEUROLOGY NEUROSURGERY AND PSYCHIATRY | 60 | 6.00 | 28 |
| 56 | Deep brain stimulation of different pedunculopontine targets in a novel rodent model of Parkinsonism | GUT NK | 2015 | JOURNAL OF NEUROSCIENCE | 58 | 6.44 | 82 |
| 57 | Subthalamic nucleus-deep brain stimulation for early motor complications in Parkinson's disease-the earlystim trial: early is not always better | MESTRE TA | 2014 | MOVEMENT DISORDERS | 58 | 5.80 | 27 |
| 58 | Fiber tractography of the axonal pathways linking the basal ganglia and cerebellum in Parkinson disease: implications for targeting in deep brain stimulation | SWEET JA | 2014 | JOURNAL OF NEUROSURGERY | 58 | 5.80 | 24 |
| 59 | Subthalamic nucleus deep brain stimulation is neuroprotective in the a53t α-synuclein Parkinson's disease rat model | MUSACCHIO T | 2017 | ANNALS OF NEUROLOGY | 57 | 8.14 | 37 |

Table 2 (Continued)

| Rank | Title | Author | Year | Journal | Total citation | Average citation by year | References |
| --- | --- | --- | --- | --- | --- | --- | --- |
| 60 | Cortical plasticity induction by pairing subthalamic nucleus deep-brain stimulation and primary motor cortical transcranial magnetic stimulation in Parkinson's disease | UDUPA K | 2016 | JOURNAL OF NEUROSCIENCE | 57 | 7.13 | 45 |
| 61 | Motor and nonmotor circuitry activation induced by subthalamic nucleus deep brain stimulation in patients with Parkinson disease: intraoperative functional magnetic resonance imaging for deep brain stimulation | KNIGHT EJ | 2015 | MAYO CLINIC PROCEEDINGS | 56 | 6.22 | 67 |
| 62 | Long-term outcomes (15 years) after subthalamic nucleus deep brain stimulation in patients with Parkinson disease | BOVE F | 2021 | NEUROLOGY | 55 | 18.33 | 41 |
| 63 | Impulse control behaviors and subthalamic deep brain stimulation in Parkinson disease | MEROLA A | 2017 | JOURNAL OF NEUROLOGY | 55 | 7.86 | 41 |
| 64 | The decision-making process leading to deep brain stimulation in men and women with Parkinson's disease - an interview study | HAMBERG K | 2014 | BMC NEUROLOGY | 54 | 5.40 | 36 |
| 65 | The impact of subthalamic deep brain stimulation on sleep-wake behavior: a prospective electrophysiological study in 50 Parkinson patients | BAUMANN-VOGEL H | 2017 | SLEEP | 53 | 7.57 | 44 |
| 66 | Tremor reduction by deep brain stimulation is associated with gamma power suppression in Parkinson's disease | BEUDEL M | 2015 | NEUROMODULATION | 53 | 5.89 | 38 |
| 67 | Neuropsychological outcomes from constant current deep brain stimulation for Parkinson's disease | TRÖSTER AI | 2017 | MOVEMENT DISORDERS | 52 | 7.43 | 47 |
| 68 | Noninvasive ultrasound deep brain stimulation for the treatment of Parkinson's disease model mouse | ZHOU H | 2019 | RESEARCH | 51 | 10.20 | 63 |

Table 2 (Continued)

| Rank | Title | Author | Year | Journal | Total citation | Average citation by year | References |
| --- | --- | --- | --- | --- | --- | --- | --- |
| 69 | An external portable device for adaptive deep brain stimulation (adbs) clinical research in advanced Parkinson's disease | ARLOTTI M | 2016 | MEDICAL ENGINEERING & PHYSICS | 51 | 6.38 | 38 |
| 70 | Directional recording of subthalamic spectral power densities in Parkinson's disease and the effect of steering deep brain stimulation | BOUR LJ | 2015 | BRAIN STIMULATION | 51 | 5.67 | 63 |
| 71 | Simulation of closed-loop deep brain stimulation control schemes for suppression of pathological beta oscillations in Parkinson's disease | FLEMING JE | 2020 | FRONTIERS IN NEUROSCIENCE | 50 | 12.50 | 115 |
| 72 | Deep brain stimulation for Parkinson's disease: defining the optimal location within the subthalamic nucleus | BOT M | 2018 | JOURNAL OF NEUROLOGY NEUROSURGERY AND PSYCHIATRY | 50 | 8.33 | 23 |
| 73 | Microelectrode arrays modified with nanocomposites for monitoring dopamine and spike firings under deep brain stimulation in rat models of Parkinson's disease | XIAO GH | 2019 | ACS SENSORS | 49 | 9.80 | 52 |
| 74 | Pyramidal tract activation due to subthalamic deep brain stimulation in Parkinson's disease | MAHLKNECHT P | 2017 | MOVEMENT DISORDERS | 49 | 7.00 | 32 |
| 75 | Deep brain stimulation of the subthalamic nucleus reestablishes neuronal information transmission in the 6-ohda rat model of Parkinsonism | DORVAL AD | 2014 | JOURNAL OF NEUROPHYSIOLOGY | 49 | 4.90 | 58 |
| 76 | Clinical implications of basic neuroscience research cortical effects of deep brain stimulation implications for pathogenesis and treatment of Parkinson disease clinical implications of basic neuroscience research | LI Q | 2014 | JAMA NEUROLOGY | 49 | 4.90 | 21 |
| 77 | Frequency-specific optogenetic deep brain stimulation of subthalamic nucleus improves Parkinsonian motor behaviors | YU CX | 2020 | JOURNAL OF NEUROSCIENCE | 48 | 12.00 | 56 |

Table 2 (Continued)

| Rank | Title | Author | Year | Journal | Total citation | Average citation by year | References |
| --- | --- | --- | --- | --- | --- | --- | --- |
| 78 | The site of stimulation moderates neuropsychiatric symptoms after subthalamic deep brain stimulation for Parkinson's disease | MOSLEY PE | 2018 | NEUROIMAGE-CLINICAL | 48 | 8.00 | 62 |
| 79 | Mechanisms underlying decision-making as revealed by deep-brain stimulation in patients with Parkinson's disease | HERZ DM | 2018 | CURRENT BIOLOGY | 47 | 7.83 | 46 |
| 80 | Low-frequency deep brain stimulation for Parkinson's disease: great expectation or false hope? | DI BIASE L | 2016 | MOVEMENT DISORDERS | 46 | 5.75 | 40 |
| 81 | Effects of deep brain stimulation on rest tremor progression in early stage Parkinson disease | HACKER ML | 2018 | NEUROLOGY | 45 | 7.50 | 41 |
| 82 | Deep brain stimulation in the caudal zona incerta versus best medical treatment in patients with Parkinson's disease: a randomised blinded evaluation | BLOMSTEDT P | 2018 | JOURNAL OF NEUROLOGY NEUROSURGERY AND PSYCHIATRY | 45 | 7.50 | 39 |
| 83 | Surviving 10 years with deep brain stimulation for Parkinson's disease -a follow-up of 79 patients | HENRIKSEN MB | 2016 | EUROPEAN JOURNAL OF NEUROLOGY | 45 | 5.63 | 39 |
| 84 | Personality changes after deep brain stimulation in Parkinson's disease | PHAM U | 2015 | ParkinsonS DISEASE | 45 | 5.00 | 30 |
| 85 | Cross-frequency coupling between gamma oscillations and deep brain stimulation frequency in Parkinson's disease | MUTHURAMAN M | 2020 | BRAIN | 44 | 11.00 | 73 |
| 86 | Cognitive and psychiatric effects of stn versus gpi deep brain stimulation in Parkinson's disease: a meta-analysis of randomized controlled trials | WANG JW | 2016 | PLOS ONE | 44 | 5.50 | 44 |
| 87 | Deep brain stimulation of the pedunculopontine nucleus area in Parkinson disease: mri-based anatomoclinical correlations and optimal target | GOETZ L | 2019 | NEUROSURGERY | 43 | 8.60 | 36 |

Table 2 (Continued)

| Rank | Title | Author | Year | Journal | Total citation | Average citation by year | References |
| --- | --- | --- | --- | --- | --- | --- | --- |
| 88 | The impact of stn deep brain stimulation on speech in individuals with Parkinson's disease: the patient's perspective | WERTHEIMER J | 2014 | ParkinsonISM & RELATED DISORDERS | 43 | 4.30 | 31 |
| 89 | Coordinate-based lead location does not predict Parkinson's disease deep brain stimulation outcome | NESTOR KA | 2014 | PLOS ONE | 43 | 4.30 | 48 |
| 90 | Cost-effectiveness of deep brain stimulation for advanced Parkinson's disease in the united states | PIETZSCH JB | 2016 | NEUROMODULATION | 42 | 5.25 | 31 |
| 91 | One-pass deep brain stimulation of dentato-rubro-thalamic tract and subthalamic nucleus for tremor-dominant or equivalent type Parkinson's disease | COENEN VA | 2016 | ACTA NEUROCHIRURGICA | 42 | 5.25 | 27 |
| 92 | Impact of mild cognitive impairment on outcome following deep brain stimulation surgery for Parkinson's disease | ABBOUD H | 2015 | ParkinsonISM & RELATED DISORDERS | 42 | 4.67 | 21 |
| 93 | Frequency-dependent effects of subthalamic deep brain stimulation on motor symptoms in Parkinson's disease: a meta-analysis of controlled trials | SU DN | 2018 | SCIENTIFIC REPORTS | 41 | 6.83 | 30 |
| 94 | Intraoperative mri for optimizing electrode placement for deep brain stimulation of the subthalamic nucleus in Parkinson disease | CUI ZQ | 2016 | JOURNAL OF NEUROSURGERY | 41 | 5.13 | 26 |
| 95 | Long-term efficacy of subthalamic nucleus deep brain stimulation in Parkinson's disease: a 5-year follow-up study in china | JIANG LL | 2015 | CHINESE MEDICAL JOURNAL | 41 | 4.56 | 23 |
| 96 | Interleaved programming of subthalamic deep brain stimulation to avoid adverse effects and preserve motor benefit in Parkinson's disease | RAMIREZ-ZAMORA A | 2015 | JOURNAL OF NEUROLOGY | 41 | 4.56 | 10 |

Table 2 (Continued)

| Rank | Title | Author | Year | Journal | Total citation | Average citation by year | References |
| --- | --- | --- | --- | --- | --- | --- | --- |
| 97 | Simultaneous low-frequency deep brain stimulation of the substantia nigra pars reticulata and high-frequency stimulation of the subthalamic nucleus to treat levodopa unresponsive freezing of gait in Parkinson's disease: a pilot study | VALLDEORIOLA F | 2019 | ParkinsonISM & RELATED DISORDERS | 40 | 8.00 | 12 |
| 98 | Bayesian adaptive dual control of deep brain stimulation in a computational model of Parkinson's disease | GRADO LL | 2018 | PLOS COMPUTATIONAL BIOLOGY | 40 | 6.67 | 64 |
| 99 | GBA-Associated associated Parkinson's disease: progression in a deep brain stimulation cohort | LYTHE V | 2017 | JOURNAL OF ParkinsonS DISEASE | 40 | 5.71 | 42 |
| 100 | Unilateral deep brain stimulation of the pedunculopontine tegmental nucleus in idiopathic Parkinson's disease: Effects on gait initiation and performance | MAZZONE P | 2014 | GAIT & POSTURE | 40 | 4.00 | 30 |
